# Reduced dynamic complexity allows structure elucidation of an excited state of KRAS^G13D^

**DOI:** 10.1038/s42003-023-04960-6

**Published:** 2023-06-02

**Authors:** Fa-An Chao, Albert H. Chan, Srisathiyanarayanan Dharmaiah, Charles D. Schwieters, Timothy H. Tran, Troy Taylor, Nitya Ramakrishnan, Dominic Esposito, Dwight V. Nissley, Frank McCormick, Dhirendra K. Simanshu, Gabriel Cornilescu

**Affiliations:** 1 NCI RAS Initiative, Cancer Research Technology Program, Frederick National Laboratory for Cancer Research, Leidos Biomedical Research, Frederick, MD 21701 USA; 2 Division of Computational Bioscience, Center for Information Technology, National Institutes of Health, Building 12A, 20892-5624 Bethesda, MD USA; 3 Helen Diller Family Comprehensive Cancer Center, University of California San Francisco, 1450 3rd Street, San Francisco, CA 94158 USA

**Keywords:** Solution-state NMR, Nanocrystallography

## Abstract

Localized dynamics of RAS, including regions distal to the nucleotide-binding site, is of high interest for elucidating the mechanisms by which RAS proteins interact with effectors and regulators and for designing inhibitors. Among several oncogenic mutants, methyl relaxation dispersion experiments reveal highly synchronized conformational dynamics in the active (GMPPNP-bound) KRAS^G13D^, which suggests an exchange between two conformational states in solution. Methyl and ^31^P NMR spectra of active KRAS^G13D^ in solution confirm a two-state ensemble interconverting on the millisecond timescale, with a major P^γ^ atom peak corresponding to the dominant State 1 conformation and a secondary peak indicating an intermediate state different from the known State 2 conformation recognized by RAS effectors. High-resolution crystal structures of active KRAS^G13D^ and KRAS^G13D^-RAF1 RBD complex provide snapshots of the State 1 and 2 conformations, respectively. We use residual dipolar couplings to solve and cross-validate the structure of the intermediate state of active KRAS^G13D^, showing a conformation distinct from those of States 1 and 2 outside the known flexible switch regions. The dynamic coupling between the conformational exchange in the effector lobe and the breathing motion in the allosteric lobe is further validated by a secondary mutation in the allosteric lobe, which affects the conformational population equilibrium.

## Abstract (web summary)

Structural analysis reveals an intermediate (excited) state of GMPPNP-bound KRAS^G13D^ which includes the formation of a new pocket in the allosteric lobe that could be exploited for developing new therapeutics against KRAS^G13D^-driven cancers.

## Introduction

RAS proteins are small GTPases, cycling between the GTP-bound form (active) and the GDP-bound form (inactive). Regulatory proteins facilitate the cycling: guanine nucleotide exchange factors (GEFs) promote conversion from the GDP-bound form to the GTP-bound form, and GTPase activating proteins (GAPs) return RAS to the GDP-bound form. RAS proteins are the central node in signaling pathways controlling cell proliferation, survival, and differentiation through interactions with various effectors, such as RAF, PI3K, and others. Oncogenic RAS mutants are the main driving factors in many lethal cancers^1^. KRAS, one of the three RAS isoforms (KRAS, HRAS, NRAS), is the most frequently mutated RAS protein in cancer patients, and G12, G13, and Q61 are three hot spots among the oncogenic mutations. These mutants sample the RAS conformational space differently. For example, the KRAS G13D mutant (KRAS^G13D^), which has a reduced oncogenic phenotype compared to G12D, was found to favor State 1 conformation (with the switch I region open). It was recently shown that KRAS^G13D^ has a stronger binding affinity to the guanine nucleotide exchange factor, Son of Sevenless (SOS), than wild-type KRAS (KRAS^WT^)^2^, and can allosterically increase the nucleotide exchange rate of KRAS at the active site more than twice of that in KRAS^WT^. On the other hand, the rate of SOS-independent nucleotide exchange for GDP-bound KRAS^G13D^ is an order of magnitude faster than that of GDP-bound KRAS^WT ^^3,4^, which could result in auto-activation by spontaneous GDP/GTP exchange. A similarly elevated SOS-independent nucleotide exchange rate was also observed for HRAS^G13D^ (15-fold compared to HRAS^WT^)^4,5^. Although KRAS^G13D^ is partially sensitive to NF1-mediated GTP hydrolysis^6^, it will be clinically important to develop a selective inhibitor targeting the oncogenic mutant KRAS^G13D^. In recent years G-quadruplex structure (G4) formation in the *KRAS* promoter^7,8^ was identified in cancer cells with effects on active oncogene expression, increased genomic instability, and telomere maintenance. This has opened yet another therapeutic avenue of finding ligands that would stabilize G4s to downregulate KRAS^9–11^.

**Fig. 1:**
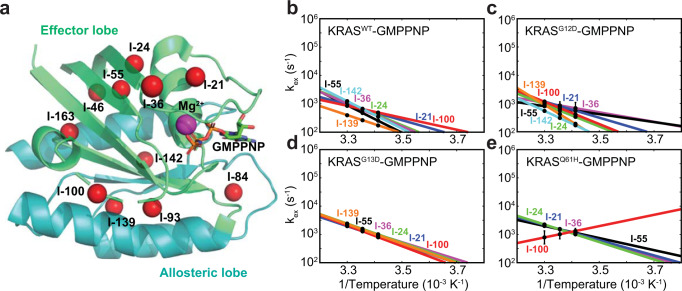
Temperature-dependent conformational dynamics of GMPPNP-bound wild type and oncogenic mutants of KRAS revealed by single-quantum ^13^C methyl CPMG experiments. **a** Methyl probes are distributed in the effector and allosteric lobes of KRAS (PDB: 6VC8). Results for KRAS^WT^, KRAS^G12D^, KRAS^G13D^, and KRAS^Q61H^ proteins with δ_1_-labeled methyl groups in isoleucine residues are plotted in panels **b,** **c,** **d**, and **e**, respectively. Each methyl group data set contains three measurements at three different temperatures (30, 25, and 20 °C) that are fitted separately, assuming a simple two-site exchange model with reaction rates (k_1_ and k_−1_) following the Arrhenius equation and a fixed chemical shift difference (Δω) at different temperatures. The errors of the fitting are reported as the standard deviations among the 10 best-fitting results out of 100 that start from randomly selected initial points. The methyl data are reported only when the amplitudes of their relaxation dispersion profiles at 25 °C are well above the experimental errors (Supplementary Fig. 1). The calculated exchange rates (k_ex_ = k_1_ + k_−1_) are plotted against temperatures. Residues I-21, I-24, I-36, I-55, I-100, I-139, and I-142 are shown in blue, green, purple, black, red, orange, and cyan, respectively.

The G-domain of RAS proteins comprises two lobes (Fig. 1a), the effector lobe (residues 1–86) and the allosteric lobe (residues 87–166). In all RAS proteins, the G-domain is followed by a C-terminal hypervariable region (residues 167–188/189) involved in plasma membrane association following isoform-dependent palmitoylation and farnesylation. The effector lobe is fully conserved across all RAS isoforms, while the allosteric lobe has 90% sequence identity among the RAS isoforms. Two conformational states in RAS proteins, detected initially by crystal structures and solution-state NMR^12,13^, correspond to an ‘open’ versus a ‘closed’ position of the dynamic Switch I relative to the bound nucleotide in the active state. They are deemed States 1 and 2 conformations, with the latter adopted in complexes with effector proteins. Various literature reviews provide a wealth of information on the available KRAS structural data giving insights into conformational dynamics from experimental data and molecular dynamics (MD) simulations^1,14–17^. In the known conformational space of KRAS, the differences between State 1 and 2 conformations in most crystal structures are highly localized to the Switch I region (residues 30–38, showing two states corresponding to the open/closed configurations) and the Switch II region (residues 59–76), which exhibit a sizeable conformational variability among various crystal structures, while the rest of the backbone is highly similar (RMSD of ~0.3–0.5 Å when both switch regions are excluded). The high similarity of the crystal structures is evident not only for the WT form but also across the deposited structures of various KRAS mutants (including G13D) and even across their GDP vs. GTP-analogs structures, which suggests this represents the protein’s ground state^18^. Although several studies on RAS proteins by solution-state NMR have shown the presence of allostery connecting the effector and allosteric lobes using chemical shift perturbations^5,19^ and relaxation dispersion experiments^20–22^, it is not clear how those observations can be translated to structural changes outside of the switch regions in KRAS in solution, which is the focus of our present NMR study. It had been shown that the dynamics of the HRAS isoform can be affected by using non-hydrolyzable GTP analogs^23^, and recent dynamic studies using GTP with HRAS^22^ and with KRAS^24^ showed that two different minor states of GTP-bound RAS can be produced under different experimental conditions. Interestingly, although HRAS and KRAS proteins have an identical primary sequence in the effector lobe, they display different structural features in the reported minor states, highlighting the complex dynamics of RAS proteins in solution. The observed functional differences among various oncogenic mutants are likely rooted in subtle conformational and dynamic differences^14^. To provide insights and inspire therapeutic strategies, it would be valuable to elucidate the solution structure of any RAS high-energy state (excited, intermediate, or transition state) relative to the well-known ground state structures (lowest energy, corresponding to existing crystal structures).

While RAS proteins are known to sample two major conformational states, States 1 and 2, previous NMR studies^19,21^ have shown that RAS proteins also exhibit many sub-states that interconvert on millisecond and microsecond (ms-μs) timescales in solution. When several excited states are present in solution, it is difficult to acquire sufficient NMR data to characterize each of them. Often, crystallography provides a single snapshot representing the ground state. To examine the complex dynamics, we explored several oncogenic mutants and detected uniquely synchronized conformational dynamics in KRAS^G13D^ bound to GMPPNP (a non-hydrolyzable GTP-analog) by methyl Carr-Purcell Meiboom-Gill (CPMG)^25^ relaxation dispersion experiments, which can reveal conformational exchanges when the exchange rates between distinct conformational states are within the detectable timescale. By recording methyl NMR spectra at low temperatures to reduce the exchange rates, we could confirm the exchange between two conformational states on the ms timescale present in both effector and allosteric lobes of GMPPNP-bound KRAS^G13D^. Furthermore, the ^31^P spectrum collected at low temperature showed a major P^γ^ atom peak corresponding to the State 1 conformation and a secondary peak indicating an intermediate state, or a transition state, different from State 2.

The solution state residual dipolar couplings (RDCs) contain information on population-weighted bond orientations of fast exchanging conformers with non-split NMR peaks, assuming Gaussian fluctuations are integrated over the entire NMR timescale of the measurement, i.e., tens of milliseconds^26,27^. Thus, the structural restraints for the intermediate state can be determined from RDCs, acquired in different alignment media, when the structure of the ground state is known. To provide ground state conformations for States 1 and 2, we solved crystal structures of GMPPNP-bound KRAS^G13D^ and KRAS^G13D^-RAF1 RBD complex at 1.2 and 2.0 Å resolutions, respectively. We then performed a population-weighted, two-state (*N* = 2) ensemble structural refinement using experimental RDC restraints and obtained a structural ensemble representing the minor GMPPNP-bound KRAS^G13D^ intermediate conformational state. The structural ensemble of the intermediate state in GMPPNP-bound KRAS^G13D^ was cross-validated by the improvement in the fit of each RDC set when iteratively excluded from the structural calculation restraints. A similarly calculated two-state structural ensemble of KRAS^WT^ could not be cross-validated by RDCs, due to the lack of synchronized dynamics. Finally, we introduced secondary mutations to further validate the structure of the intermediate state and the dynamic coupling between the effector and allosteric lobes. Since wild-type and mutant KRAS in complex with effectors adopts the conformational State 2 in all crystal structures determined to date, presumably by both conformational selection and induced fit, the structure of an intermediate state could likely shed light into these interactions, with potential relevance for drug design.

In summary, we detected an intermediate state of active KRAS^G13D^ by NMR relaxation dispersion and chemical shifts. We used RDCs to solve and cross-validate this intermediate state structure with a conformation distinct from those of States 1 and 2 outside the known flexible switch regions. We obtained high quality representations of States 1 and 2 by crystallography. The dynamic coupling between the effector and allosteric lobes is further validated by a secondary mutation in the allosteric lobe, which affects the conformational population equilibrium.

## Results

### Synchronized conformational dynamics in GMPPNP-bound KRAS^G13D^ revealed by relaxation dispersion experiments

Although the dynamics of RAS proteins had been extensively studied in solution by ^31^P NMR, which reports two major conformational states in the effector lobe (Fig. 1a), recent studies^19,21^ demonstrated the presence of global dynamics as well as local dynamics throughout the whole protein (both effector and allosteric lobes) on the ms-μs timescale. We also showed that altered conformational dynamics on the ms-μs timescale subtly tune the biological functions of mutant KRAS using sensitivity-enhanced methyl relaxation dispersion experiments^21^. While investigating the conformational dynamics of oncogenic mutants using this method (Fig. 1b–e and Supplementary Fig. 1), we found that GMPPNP-bound KRAS^G13D^ displays a rare synchronized conformational dynamics behavior connecting the effector and allosteric lobes. The conformational exchange rates are reduced relative to GDP-bound KRAS^G13D^, which shows fast and diverse conformational dynamics (Supplementary Fig. 2). This overall trend of reduced conformational exchange rates of KRAS^G13D^ in the presence of γ phosphate is similar to that of KRAS^WT^ (Supplementary Fig. 2). Still, the conformational dynamics of GMPPNP-bound KRAS^G13D^ is surprisingly more synchronized than that of other KRAS oncogenic mutants (Fig. 1b–e). In our analysis, although we could globally fit the relaxation dispersion data of GMPPNP-bound- KRAS^G13D^ with a simple two-site exchange model at 25 °C, the data were insufficient to disentangle the population distribution and chemical shift differences between the two dominant conformational states (Supplementary Fig. 3a). However, by combining all relaxation dispersion data from different temperatures, we could determine that the minor state represents ~15% of the population but with a significantly elevated χ^2^ when assuming the simple two-site exchange and Arrhenius equation (Supplementary Fig. 3b). The elevated χ^2^ suggests that this assumption is incompatible with our experimental data; therefore, our relaxation dispersion data suggest fast exchanges among substates within the major conformational states on the ms-μs timescale may exist. At the same time, by investigating the amide relaxation dispersion data, we also observed the ms-μs backbone conformational dynamics throughout the G-domain of GMPPNP-bound KRAS^G13D^ (Supplementary Fig. 4). Thus, the combined relaxation dispersion data confirm that the ms-μs conformational dynamics are not restricted to the effector lobe of KRAS proteins, which contains the switch regions but are distributed throughout the G-domain.

### The two-state conformational exchange of GMPPNP-bound KRAS^G13D^ on the millisecond timescale revealed by spectral analysis

**Fig. 2:**
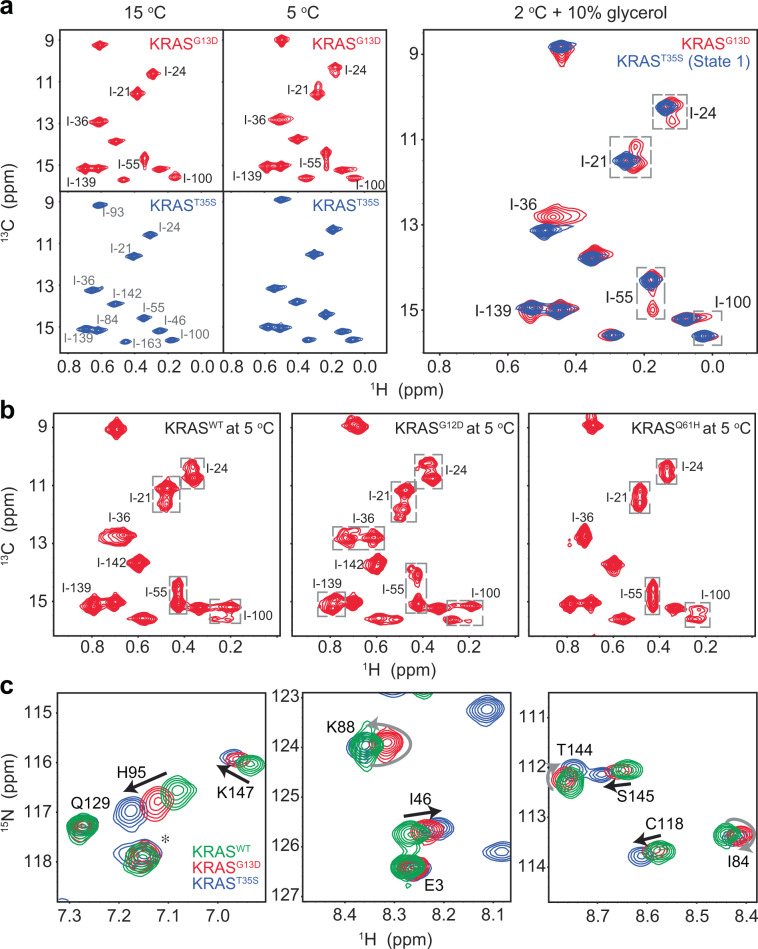
Two-site conformational exchange in GMPPNP-bound KRAS^G13D^ on the millisecond timescale observed by spectral analysis. **a** The two-state exchange on the ms timescale revealed by methyl-TROSY spectra of KRAS^G13D^ and KRAS^T35S^ by lowering the temperature. In the right panel, methyl signals of KRAS^G13D^ and KRAS^T35S^ are overlaid, and the methyl resonances in KRAS^G13D^ with detectable relaxation dispersion profiles (Supplementary Fig. 1) are labeled. The assignments of all methyl resonances are shown in the KRAS^T35S^ 15 °C spectrum in panel **a**. GMPPNP-bound KRAS^G13D^ and KRAS^T35S^ are shown in red and blue, respectively. **b** The complex local dynamics on the ms timescale revealed by methyl-TROSY spectra of KRAS^WT^, KRAS^G12D^, and KRAS^Q61H^ at 5 °C. The methyl resonances with detectable relaxation dispersion profiles (Supplementary Fig. 1) are labeled. **c** Overlaid amide spectral regions of GMPPNP-bound KRAS^G13D^, KRAS^T35S^, and KRAS^WT^, at 25 °C, are shown in red, blue, and green, respectively. All assigned resonances are labeled, an unassigned arginine N^η^H^η^ side-chain resonance is marked by the asterisk.

**Fig. 3:**
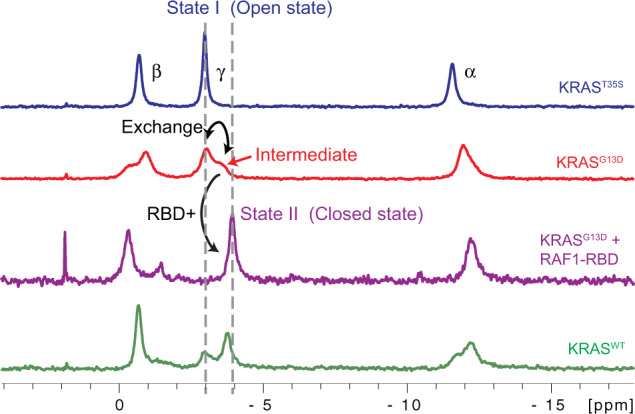
An intermediate state (or a transition state) of the GMPPNP-bound KRAS^G13D^. An intermediate state distinct from States 1 and 2 is revealed in the ^31^P NMR spectra of the GMPPNP-bound KRAS^G13D^ at 5 °C.

Thermodynamically, temperature change has little effect on the enthalpic term (structures of the conformational states). In contrast, it substantially impacts the entropic term (exchanges among different conformational states). Because the global analysis of our CPMG data on GMPPNP-bound KRAS^G13D^ could not independently validate the two-state exchange model, we further investigated the lineshape changes of methyl resonances when slowing the exchange rates by lowering the temperature (Fig. 2a). Similar lineshape analysis was used to investigate ms conformational dynamics in previous ^31^P NMR studies^28,29^. Indeed, we could observe methyl resonance splitting starting at 5 °C for KRAS^G13D^ but not for KRAS^T35S^ (a KRAS mutant known to stabilize State 1) (Fig. 2a). After adding 10% glycerol to increase viscosity and lowering the temperature to 2 °C to further decrease the conformational exchange rate, complete separation between the major peak (representing the major conformational state) and the minor peak (representing the minor conformational state) could be observed for the methyl resonance of I-55 (Fig. 2a). The dominant population is in the State 1 conformation (with peaks matching those of GMPPNP-bound KRAS^T35S^), and the peak heights suggest that the minor state represents ~30% of the population (Fig. 2a). At low temperatures, all methyl groups with relaxation dispersion profiles in GMPPNP-bound KRAS^G13D^ (Supplementary Fig. 1c) show complete or partial resonance splitting into two peaks or resonance broadening, as expected for a range of chemical shift differences corresponding to the two interconverting conformational states (Fig. 2a). Combined with our relaxation dispersion data, this spectral analysis confirmed the existence of a synchronized two-state conformational exchange on the ms timescale spanning both lobes of GMPPNP-bound KRAS^G13D^. In contrast, all methyl spectra of GMPPNP-bound KRAS^WT^, KRAS^G12D^, and KRAS^Q61H^, at 5 °C, showed more complex dynamic behaviors on the ms timescale (Fig. 2b), as expected from our data analysis of CPMG experiments (Fig. 1b–e). In the case of KRAS^WT^, the methyl resonances from I-21, I-24, and I-55 in the effector lobe split into two major peaks, while the methyl resonance from I-100 in the allosteric lobe split into one major peak and two minor peaks. In the case of KRAS^G12D^, the methyl resonances from I-21, I-24, and I-55 in the effector lobe split into two major peaks and one minor peak, while the methyl resonance from I-139 in the allosteric lobe split into two major peaks. The resonance from I-100 in the same lobe splits into two major peaks and two minor peaks. In the case of KRAS^Q61H^, the methyl resonances from I-21, I24, and I-55 in the effector lobe split into two major peaks. In comparison, the methyl resonance from I-100 in the allosteric lobe splits into two broad peaks, indicating more complex ms dynamics in the allosteric lobe. At 25 °C, we also examined the amide chemical shifts of GMPPNP-bound KRAS^WT^, KRAS^G13D^, and KRAS^T35S^. We observed that some resonances (but not all) show a linear path following the increasing population of State 1 conformation in mutant KRAS (Fig. 2c). The conformational state populations in mutant KRAS were determined by the ^31^P spectra (Fig. 3). As reported previously^28,29^, similar resonance splitting was also observed in the ^31^P spectra of nucleotide-bound RAS proteins at low temperatures (Fig. 3). Based on the ^31^P spectra, the population of State 1 conformation is ~35% in KRAS^WT^, ~70% in KRAS^G13D^, and ~100% in KRAS^T35S^. However, in our amide HSQC spectra (Fig. 2c), we also observed that many other resonances do not show a linear path following an increasing population of the State 1 conformation. Since the amide chemical shifts of KRAS^T35S^ represent the State 1 conformation and those of KRAS^WT^ represent the structural averaging between the State 1 and 2 conformations, this suggests that the conformation of the minor state in GMPPNP-bound KRAS^G13D^ is not simply an average conformation between the State 1 and 2 conformations. While analyzing ^31^P NMR spectra of the nucleotide γ phosphate in GMPPNP-bound KRAS proteins at 5 °C, we again observed two conformational states of GMPPNP-bound KRAS^G13D^ in solution on the millisecond timescale. The major population is in the State 1 conformation, and the minor one is in an intermediate state (Fig. 3), likely with a semi-open state of the Switch I region. The peak heights suggested that the minor state in GMPPNP-bound KRAS^G13D^ represents ~30% of the population, in agreement with the intensity ratio of the split methyl resonances at low temperatures (Fig. 2a). The existence of an intermediate state in GMPPNP-bound KRAS^G13D^ supports the proposed mechanism of the interaction between RAS proteins and RAF1-RBD in a previous transient kinetic analysis of RAS proteins^30^, where an initial rapid equilibrium step (conformational selection) is followed by an isomerization reaction (induced fit).

### Crystal structures of GMPPNP-bound KRAS^G13D^ and KRAS^G13D^-RAF1 RBD represent the State 1 and 2 conformations, respectively

**Fig. 4:**
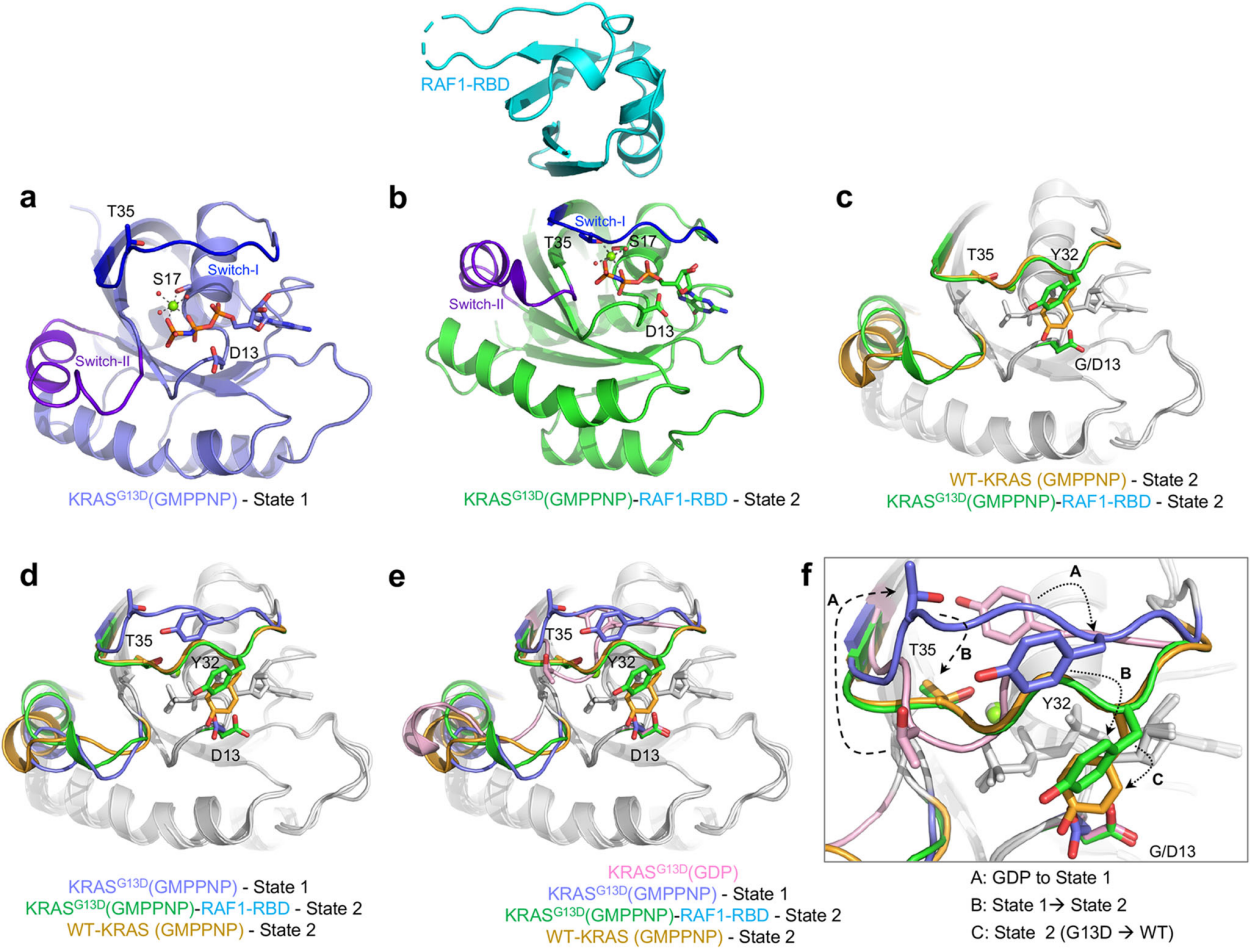
Crystal structures of GMPPNP-bound KRAS^G13D^ in State 1 and State 2 conformations and conformational analysis of the Switch I region. **a** The overall structure of GMPPNP-bound KRAS^G13D^ in State 1 conformation. KRAS^G13D^ (PDB 8EBZ) is colored light blue, with the Switch I and II regions highlighted in blue and purple, respectively. GMPPNP is shown as sticks, and Mg^2+^ (green) and water (red) molecules are shown as spheres. **b** The overall structure of GMPPNP-bound KRAS^G13D^ (green; State 2 conformation) in complex with RAF1-RBD (cyan) (PDB 8EPW). **c** Structural superposition of WT (orange; PDB 6GOD) and G13D (green; PDB 8EPW) mutant of KRAS in State 2 conformation showing differences in switch regions. Side-chain atoms of Y32 and T35 are shown to highlight conformational differences in these structures. **d** Structural superposition of KRAS^WT^ (State 2; PDB 6GOD) with KRAS^G13D^ in State 1 (PDB 8EBZ) and State 2 (PDB 8EPW) conformations showing differences in switch regions. **e** Structural comparison of KRAS^WT^ (State 2; PDB 6GOD) with KRAS^G13D^ in GDP (light magenta; PDB 4TQA) and GMPPNP-bound (PDB 8EBZ, 8EPW) forms showing differences in the switch regions. **f** Enlarged view of the Switch I region from panel E shows the transition of the Switch I region from GDP to State 1, State 1 to State 2, and State 2 conformational similarities (T35) and differences (Y32) between WT and G13D mutant of KRAS.

To gain detailed insights into the State 1 and 2 conformations of active KRAS^G13D^, including the Switch regions, we solved high-resolution crystal structures of GMPPNP-bound KRAS^G13D^ and KRAS^G13D^ in complex with RAF1 RBD at 1.2 Å and 2.0 Å, respectively (Table 1). The electron density associated with GMPPNP, magnesium, and switch regions was unambiguous in the high-resolution maps. This contrasts with the recently reported 3.4 Å resolution structure of GMPPNP-bound KRAS^G13D^ (PDB: 6E6F; RMSD: 0.61 Å), in which the flexible Switch II could not be modeled^4^. In the GMPPNP-bound KRAS^G13D^ structure, Switch I is in State 1, since it moves away from the GMPPNP, and T35 does not contact the Mg^2+^ ion (Fig. 4a). This Switch I conformation in this structure is different from the lower resolution structure (PDB: 6E6F) despite both being in the State 1 conformation (the dynamic Switch I is captured in two different snapshots as observed in two different crystal forms) and is clearly different from the State 2 conformation of KRAS^G13D^ bound to RAF1-RBD (Fig. 4b). The overall structure of the KRAS^G13D^-RAF1-RBD complex is similar to the GMPPNP-bound KRAS^WT^ (PDB: 6GOD) and KRAS^WT^-RAF1-RBD (PDB: 6VJJ) structures previously reported in State 2 conformation, except that Y32 in the Switch I region occupies a different rotameric conformation due to steric clash with D13 (Fig. 4c). The difference in Switch II is likely due to different crystal packing interactions and is not believed to be associated with the G13D mutation, different states of Switch I, or the presence of RAF1-RBD. The sidechain of D13 in GMPPNP-bound KRAS^G13D^ points toward Switch II (χ_1_: −51.3°), which is similar to that in the lower resolution structure (PDB: 6E6F; χ_1_: −62.9°) but opposite to that in the GDP-bound structure (PDB: 4TQA; χ_1_: −148.7°) or the RAF1-RBD-bound structure (χ_1_: −149.6°)^3^. This suggests that the D13 side chain is flexible and samples two rotameric conformations. In all cases, the D13 side chain interacts with a positively charged moiety, such as K117, or with a Mg^2+^ ion in the solvent introduced by the crystallization condition. A structural comparison of GMPPNP-bound KRAS^WT^ in the State 2 conformation with KRAS^G13D^ in the GDP-bound conformation and GMPPNP-bound State 1 and 2 conformations shows four different snapshots of the Switch I region (Fig. 4d–f). Sidechain atoms of T35 and Y32 transition in opposite directions in these snapshots, illustrating how the conformation ensembles of the Switch I region change from inactive to active (State 1) and then from State 1 to State 2 conformations. The conformation of the T35 side chain is maintained in the State 2 conformation of WT and G13D mutant of KRAS; however, the Y32 side chain occupies a different rotameric conformation due to a steric clash with D13 in the KRAS^G13D^ structure (Fig. 4f).

**Table 1:** Crystallographic data collection and refinement statistics.

	GMPPNP-bound KRAS^G13D^ (8EBZ)	KRAS^G13D^-RAF1-RBD (8EPW)
Data collection		
Space group	C 1 2 1	P 2 21 21
Cell dimensions		
*a*, *b*, *c* (Å)	120.93, 38.23, 36.32	44.48, 66.8, 71.38
α, β, γ (°)	90, 103.28, 90	90, 90, 90
Resolution (Å)	35.35-1.2 (1.24-1.2)	37.75-2.0 (2.07-2.0)
*R*_sym_ or *R*_merge_	0.044 (0.184)	0.103 (0.598)
*I* /σ*I*	20.33 (7.91)	14.31 (4.01)
Completeness (%)	95.30 (89.60)	99.90 (99.86)
Redundancy	5.7 (5.6)	8.7 (8.9)
Refinement		
Resolution (Å)	35.35-1.2	37.75-2.0
No. reflections	48364 (4539)	14915 (1459)
*R*_work_ / *R*_free_	0.1591 (0.1861)/0.1709 (0.2121)	0.1947 (0.2899)/0.2528 (0.3752)
No. atoms		
Protein	1400	1911
Ligand/ion	36	33
Water	186	61
*B*-factors		
Protein	19.61	38.19
Ligand/ion	10.33	26.03
Water	30.44	39.56
R.m.s. deviations		
Bond lengths (Å)	0.005	0.007
Bond angles (°)	0.802	0.946

Values in parentheses are for highest-resolution shell.

One crystal was used for data collection for each structure.

### Structural analysis of GMPPNP-bound KRAS^G13D^ by RDCs

All RAS crystal structures show consistent conformational variability exclusively in the flexible Switch I and II regions due to variations in crystal packing. Our relaxation dispersion data and NMR chemical shift analysis demonstrated the presence of conformational exchange between two dominant states outside the flexible switch regions because our methyl probes and measured RDCs span the entire G-domain (i.e., throughout both effector and allosteric lobes). We measured residual dipolar couplings (RDCs) to elucidate the structural features of the excited state in solution distal from the Switch I and II regions. Since experimental RDCs report time-averaged conformational states^31^, we examined this two-state exchange model of GMPPNP-bound KRAS^G13D^ in solution by measuring RDCs of various bond vectors in two alignment media (Pf1 and bicelles). Although the ^1^D_NH_ and ^1^D_CαHα_ RDCs of the GMPPNP-bound KRAS^G13D^, measured in both Pf1 and bicelles, aligned well with high-resolution crystal structures, the best fit RMSD was 2-3 times larger than the corresponding RDC measurement precision (Supplementary Figs. 5 and 6). This suggested that the crystal structures do not completely represent the solution state of KRAS^G13D^ and led us to generate an *N* = 2 ensemble of solution structure models that would fit the RDC restraints within (but not tighter than) the experimental precision, as shown in Supplementary Fig. 5. Therefore, the resulting structural ensemble refined by RDCs is a more accurate representation of the conformational states sampled by GMPPNP-bound KRAS^G13D^ in solution.

### The structure of the intermediate state of GMPPNP-bound KRAS^G13D^ and its validation

**Fig. 5:**
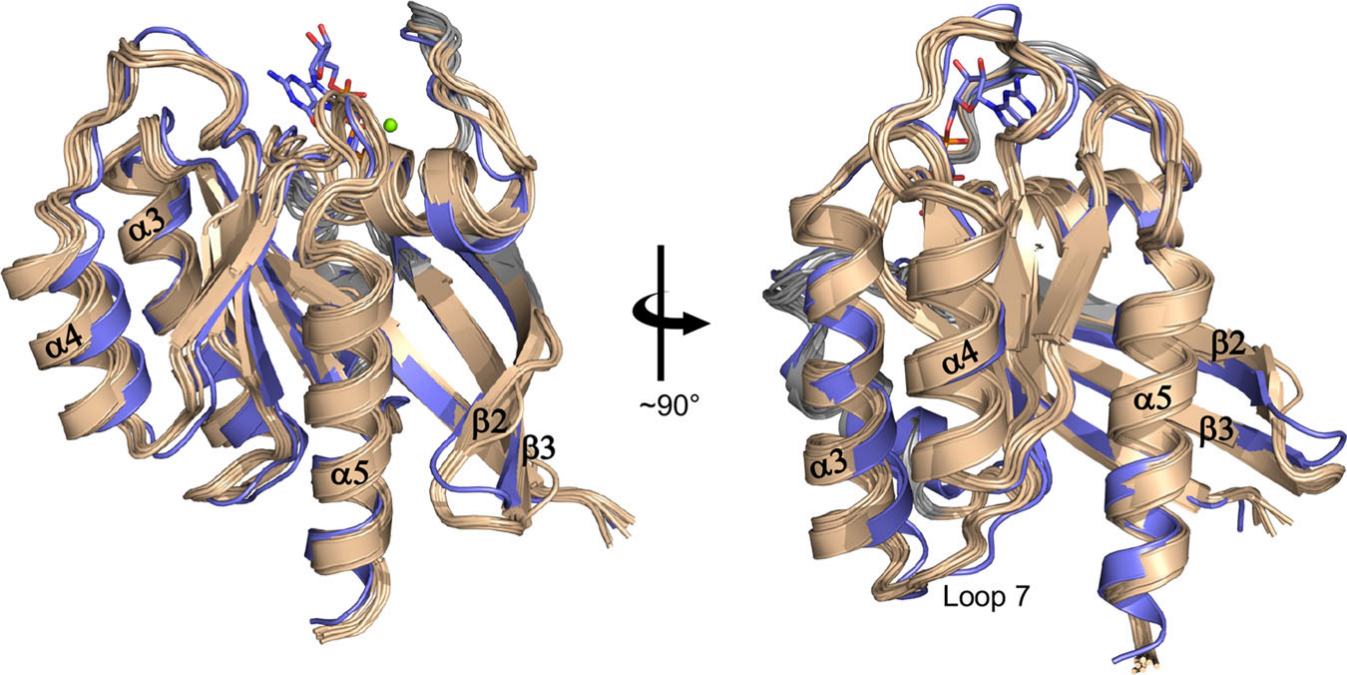
RDC refined structures. The intermediate state of the GMPPNP-bound KRAS^G13D^ (minor 30% conformer, colored wheat) overlaid to the State 1 conformation (major 70% conformer, crystal structure, colored blue).

The experimental RDCs of GMPPNP-bound KRAS^G13D^ could not be measured for most residues in the switch regions due to the complete exchange broadening of amide resonances with large chemical shift differences. Therefore, we focused on refining the backbone of the GMPPNP-bound KRAS^G13D^ using the observable residues to obtain the intermediate state conformation. The refinement protocol fixed the major conformer to the State 1 conformation (represented by the GMPPNP-bound KRAS^G13D^ crystal structure), as indicated by the ^31^P spectra. We calculated RDC-refined structures of the GMPPNP-bound KRAS^G13D^ starting from the corresponding crystal structures (State 1 conformation) and allowing a maximum 3 Å displacement of the heavy atom coordinates under simulated annealing using all experimental RDC restraints. We carefully adjusted the relative force constants of the RDC energy terms so that the calculated structures would fit the RDCs within the experimental errors (less than 1 Hz for NH and less than 2 Hz for C^α^H^α^, Supplementary Fig. 5), but without overfitting. Additionally, backbone dihedral angle restraints obtained from NMR chemical shifts using the Talos-N^32^ program were included in the structure calculations. We also performed a grid search by repeating the *N* = 2 ensemble refinement and RDC cross-validation with various relative population ratios and found an optimal ratio of 30:70, in agreement with the ^31^P and methyl spectra at 5 °C (Figs. 2 and 3). This population ratio resulted in the lowest dihedral angle potential energies, while the other energy terms (except the RDC term) remained essentially unchanged. Separate structural calculations to cross-validate the obtained intermediate state of GMPPNP-bound KRAS^G13D^ were performed by excluding each RDC set to calculate the R^free^ quality factor^33^. This factor quantitates the agreement between each experimental RDC set and the corresponding RDC values back-calculated from the structural ensemble obtained when that set was not used as a structural restraint, as shown in Supplementary Fig. 6. Notably, the calculated conformations representing the intermediate state satisfy the RDC cross-validation tests, as shown in Supplementary Figs. 6 and 7. These R^free^ values for the *N* = 2 ensemble (weighted RDC fit over both major and minor conformations) exhibit small but consistent improvements over the R values of the major conformation only (i.e., ground state, represented by the crystal structure of State 1). The *N* = 2 cross-validation improvements are more pronounced for the RDCs with smaller relative experimental errors, i.e., NH and C^α^H^α^ in Pf1 and NH in bicelles, but less pronounced for C^α^H^α^ in bicelles due to a slight instability in the bicelles sample, which began to slowly hydrolyze during the experiment (the buffer pH was above neutral), and for the much smaller ^1^D_NC’_ and ^2^D_NC’_ couplings. When selecting only the residues showing the most significant changes in the spatial orientation of the RDC vectors (i.e., N-H, Cα-Hα, N-C’_i_, N-C’_i-1_ of residues 7, 13–15, 23–29, 43–54, 60–63, 73–81, 86, 92–95, 105–113, 120–124, 137–140, 144–150), the cross-validation improvements in the R^free^ quality factor (or in the RMSD of the fit) are easier to visualize (Supplementary Fig. 7). Thus, the statistical improvement in the *N* = 2 ensemble refinement of GMPPNP-bound KRAS^G13D^ over the starting crystal structure validates the existence of an excited state in solution. The intermediate state (Fig. 5) is represented by the ten lowest energy structures (out of 100 calculated) and has an RMSD of 1.0 Å from the mean structure. The pairwise RMSDs between these and the ground state (i.e., the starting crystal structure) are between 1.0 and 1.3 Å (with no significant displacements of the switch regions, as discussed above). The average backbone RMSD values between the ground state (crystal structure) and the excited state (refined structural ensemble) are shown in Supplementary Fig. 8.

### Structural analysis of GMPPNP-bound KRAS^WT^ by RDCs

To further validate the above results, we applied the same approach to GMPPNP-bound KRAS^WT^, which has more complex conformational dynamics shown by relaxation dispersion data (Fig. 1a). We similarly examined the *N* = 2 ensemble refinement of the GMPPNP-bound KRAS^WT^ with RDCs (using NH and C^α^H^α^ measured in Pf1 and bicelles) and found an optimal ratio of approximately 60:40. Although the result agreed with the ^31^P spectrum (Fig. 3), the RDC cross-validation showed no significant improvement over the fit to the starting high-resolution crystal structure in the State 2 conformation (Supplementary Table 1). The calculated minor state of GMPPNP-bound KRAS^WT^ showed similar structural displacements from the starting crystal structure as did the excited state of KRAS^G13D^. Still, only the GMPPNP-bound KRAS^G13D^ structures exhibited statistically significant R^free^ improvements of the RDCs. When comparing directly their corresponding experimental RDCs (NH and C^α^H^α^ measured in Pf1 and bicelles), the most prominent outliers, having consistent RDC differences 1.5 times larger than the pairwise RMSD (mapped on the structure in Supplementary Fig. 9), correlated well with the regions of the GMPPNP-bound KRAS^G13D^ intermediate state showing displacements from the ground state (Supplementary Figs. 8 and 9). This suggests that a simple population-weighted average of just two conformational states represents more accurately the average solution structure of KRAS^G13D^ than that of KRAS^WT^. For GMPPNP-bound KRAS^WT^, a similar *N* = 2 ensemble refined by RDCs likely represents an oversimplification of a more complex distribution of intermediate states. Therefore, our data suggest that the KRAS^WT^ crystal structures might already accurately represent the average of its complex dynamic fluctuations in solution outside the switch regions, while this was not the case for KRAS^G13D^.

### Validation of the synchronized motion and the structure of the intermediate (excited) state in GMPPNP-bound KRAS^G13D^

**Fig. 6:**
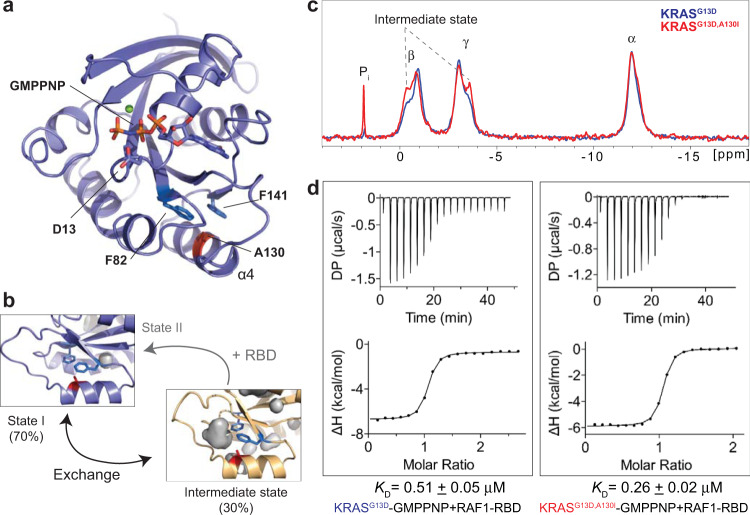
Perturbation of the population equilibrium and effector binding affinity through secondary mutations in the allosteric lobe of GMPPNP-bound KRAS^G13D^. **a** The displaced conformation of the α4 helix in the allosteric lobe of the intermediate state is shown on the crystal structure (PDB: 8EBZ). **b** In the allosteric lobe, a small hydrophobic pocket near A130 appears in the RDC-refined structure of the excited state. **c** The introduction of the secondary mutation A130I, with a larger hydrophobic sidechain, increases the population of the intermediate state as monitored by nucleotide ^31^P NMR spectra. **d** Binding affinities between GMPPNP-bound KRAS mutants and RAF1-RBD obtained by ITC measurements. The error in the *K*_D_ values corresponds to the standard error of fitting of a singlicate dataset with 19 injections.

We introduced secondary mutations in the allosteric lobe to confirm the existence of the synchronized motion and the intermediate state in GMPPNP-bound KRAS^G13D^. These mutations could cause structural perturbations in the effector lobe and shift the population equilibrium, which could be monitored by the changes in the nucleotide ^31^P NMR spectra. Close inspection of the RDC-refined structure of the intermediate state in the allosteric lobe revealed a hydrophobic cavity near residue A130, which is present in all conformers of the calculated ensemble (Fig. 6a, b). Four mutations (A130V, A130L, A130I, and A130F) with varying sizes of the hydrophobic sidechains were selected and introduced in KRAS^G13D^ to favor the formation of the cavity. All the double mutants were found to be less stable than KRAS^G13D^. Due to the low solubility of KRAS^G13D,A130F^, only three out of four double mutants could be further analyzed. In the most stable double mutant KRAS^G13D,A130I^, we found that the slight population shift (~10%) induced by a second mutation A130I (Fig. 6c) resulted in a two-fold increase in the binding affinity to RAF1-RBD compared with the single mutant KRAS^G13D^ (Fig. 6d). To confirm the relatively small differences in the high-affinity binding of KRAS mutants to RAF1-RBD (Fig. 6d), we repeated the ITC experiments on KRAS^G13D^ and KRAS^G13D,A130I^ with a higher salt concentration buffer to observe the change in titration curves caused by weakening the interactions between KRAS mutants and RAF1-RBD. Indeed, the higher salt concentration did reduce the binding strength of KRAS to RAF1-RBD but maintained the two-fold increase in the binding affinity to RAF1-RBD for the KRAS^G13D,A130I^ double mutant over that of the KRAS^G13D^ mutant (Supplementary Fig. 10). The other double mutants, KRAS^G13D,A130V^ and KRAS^G13D,A130L^, with almost identical population ratios to KRAS^G13D,A130I^ (Supplementary Fig. 11), show roughly similar binding affinities to RAF1-RBD (Supplementary Fig. 10). Consistent with our previous study^21^, the population distribution per se cannot determine the binding affinity of KRAS mutants to RAF1-RBD, and the contribution of the entropic term likely accounts for the variations in *K*_D_. In the background of the G13D mutation, the binding between KRAS mutants and RAF1-RBD is enthalpically driven under our experimental conditions, unlike that of KRAS^WT^ (Supplementary Table 2). These results confirm the dynamic coupling between the effector and allosteric lobes and validate the RDC-refined structure of the intermediate state of GMPPNP-bound KRAS^G13D^.

## Discussion

Proteins function often involves an exchange between multiple conformations that are sparsely populated and occur transiently. If the timescale of the conformational exchange is on the order of ms-μs, determining these relevant conformational states is a complex endeavor, especially when it is difficult to trap them by mutations or interactions with ligands, or when the exchange rates are not sufficiently slow to result in distinct chemical shifts for each conformation. Here we describe a novel approach to identify and elucidate an excited conformation in the presence of complex ms-μs conformational dynamics of RAS proteins. Since the dominant State 1 of GMPPNP-bound KRAS^G13D^ is not a single conformational state on the ms-μs timescale^21^, accurate chemical shift difference (Δω) values cannot be extracted from relaxation dispersion experiments. Consequently, previous NMR approaches^34–36^ to determine the structure of a sparsely populated excited state in solution using accurate determination of Δω values do not apply to this case. Even if the CPMG data cannot be globally fitted to provide detailed dynamic information, our data analysis identified a synchronized motion in the GMPPNP-bound oncogenic mutant KRAS^G13D^. Although two minor states and the conformational exchanges involved in intrinsic nucleotide exchange have been revealed by GTP-bound RAS proteins^22,24^, the results of this work and our previous study^21^ demonstrate the plasticity of KRAS proteins when interacting with their effectors instead. Using a high-resolution crystal structure and multiple sets of residual dipolar couplings, we determined a synchronized ‘breathing’ motion spanning subtle structural changes distributed through this oncogenic mutant’s G-domain (Fig. 5 and Supplementary Fig. 9), which couples with the conformational exchange in Switch I region (Figs. 1, 3, and 5). Finally, we introduced a series of additional mutations at A130 to validate the presence of a small hydrophobic pocket in the allosteric lobe by monitoring the population shifts in the effector lobe using ^31^P NMR spectra (Fig. 6c and Supplementary Fig. 11). Even though all these double mutants of KRAS display similar conformational population distribution, they have different binding affinities to the RAF1-RBD effector, as shown by ITC data (Supplementary Fig. 10). We observed a similar situation in our previous study^21^, where three different mutations stabilizing the State I conformation in KRAS displayed different dynamics coupled with different binding affinities to RAF1-RBD. The result agrees with an earlier study of the same mutations in HRAS^37^. Our ITC data and previous results^21,37^ suggest that the population distribution of RAS conformations in solution is not the sole factor affecting the binding of RAS proteins to the effectors. On the other hand, our ^31^P spectra on KRAS^G13D^ (Fig. 3) and a previous transient kinetic study^30^ support that the binding of RAS proteins to their effectors involves both conformational selection and induced fit. The induced fit from the intermediate state to the bound state and additional unpredicted entropic contributions could explain the variation in the binding affinities of different KRAS double mutants to RAF1-RBD (Supplementary Fig. 10).

The statistical improvement in the RDC cross-validation tests observed only for the GMPPNP-bound KRAS^G13D^ shows that complex dynamics do not average out the structural information of its excited state on the ms-μs timescale. In the GMPPNP-bound KRAS^G13D^, a consistent deviation between the ground state and the excited state can be observed in the allosteric lobe (Fig. 5), which has not been previously reported due to the lower resolution of the solved structures. In the RDC restrained model, the slight departures of β5 and β6 strands of the excited state relative to the ground state result in a displaced α4 helix away from the ground state (crystal structure) position.

In our study, the I46-D47-G48 (β2-β3 hairpin) amides are broadened in the NMR spectra of GMPPNP-bound KRAS^G13D^ and KRAS^WT^. This motif exhibits a displaced conformation in the minor intermediate state (Fig. 5). Conformational variation of this β2-β3 hairpin has also been observed in various crystal structures, including some determined at high pressure^38^. Moreover, MD simulations have detected correlated fluctuations of these β2-β3 strands, located between the Switch I and Switch II regions in active KRAS, with the hairpin driving Y157 (α5 helix) motions^39^. Lastly, the β2–β3 and α5 were deemed to form a conformational switch in HRAS, with mutations in D47-E49 causing hyperactive RAS^40^ and, indeed, the G48A mutation was detected in a patient with lung adenocarcinoma^40^. Another region showing a different minor conformation relative to the ground state is α3, which exhibits a reduced bend at H95. This straighter α3 helix and the displaced Loop L7 (Fig. 5) correspond to the α3-L7 motif, which, together with both switch regions, is involved in the HRAS binding of GEFs^41^. Conversely, a slightly more bent α3 helix is observed in a KRAS^G12D,K104Q^ mutant, which causes an allosteric effect on the stability of the α2 helix in Switch II when compared to KRAS^WT ^^42^. Furthermore, fluctuation in this α3-L7 motif was found to drive Switch I and II fluctuations in KRAS-GTP in MD simulations^39^. The structural differences observed in the minor state in the α4 helix, the straighter α3 helix and displaced loop 7, the β2-β3 hairpin that wraps around α5, the minor changes in β strand positions and β-sheet twist, together with the less compact packing of the helices in the allosteric lobe (when compared to the crystal structures in State 1/State 2 conformations), likely contribute to the observed functional differences of GMPPNP-bound KRAS^G13D^ vs. GMPPNP-bound KRAS^WT^.

In summary, our methyl relaxation dispersion experiments revealed highly synchronized conformational dynamics in the active KRAS^G13D^. Our high-resolution crystal structures provide snapshots of this mutant in State 1 and 2 conformations. Using RDCs and a population-weighted two-state ensemble refinement method, we solved the structure of the intermediate state, showing regions of structural fluctuations with functional relevance. This excited (intermediate) state, distinct from the known dominant States 1 and 2 in KRAS^WT^ and its G12D and Q61H mutants, is validated by three pieces of evidence. Firstly, the consistent improvement in the RDC cross-validation of the KRAS^G13D^ two-state ensemble comprising State 1 and this distinct excited state (Supplementary Figs. 6 and 7) in contrast with the KRAS^WT^ test case that uses States 1 and 2, which shows no similar improvement (Supplementary Table 1). This is because only KRAS^G13D^ is a simple two-conformation state system, while KRAS^WT^ is a more complex multi-state system with less synchronized conformational motion. Secondly, the distinct chemical shift of the ^31^Pγ signal of KRAS^G13D^ in Fig. 3 confirms that the nucleotide-binding pocket samples an excited conformation different from the known States 1 and 2 in KRAS^WT^ and most of its mutants. Thirdly, the non-linearity of the trajectory of some KRAS^G13D^ amide chemical shifts when superimposed on the spectra of KRAS^T35S^ and KRAS^WT^ (representing respectively the State 1 and a conformational average of States 1 and 2, Fig. 2c) confirms that these differences occur throughout the rigid backbone of the protein (distal from the Switch regions). The structural analysis of the intermediate (excited) state in GMPPNP-bound KRAS^G13D^ suggests the formation of a new pocket in the allosteric lobe, which could be exploited for developing new therapeutics against KRAS^G13D^-driven cancers. Our approach can be applied to other proteins with similar dynamic behavior.

## Materials and methods

### DNA for protein production

Genes for protein expression were generated from DNA constructs initially synthesized as Gateway Entry clones (ATUM, Newark, CA). Constructs consisted of *E. coli* gene-optimized fragments containing an upstream tobacco etch virus (TEV) protease site (ENLYFQ/G) followed by the coding sequence of human KRAS4b(1-169) or human RAF1(52-131). Entry clones were transferred to an *E. coli* destination vector containing an amino-terminal His6-MBP (pDest-566, Addgene #11517) tag by Gateway LR recombination (Thermo Scientific, Waltham, MA). Constructs generated were:

R989-X02-566: His6-MBP-tev-G-Hs.KRAS4b(1-169) [Addgene:159539]

R989-X09-566: His6-MBP-tev-G-Hs.KRAS4b(1-169) G12D [Addgene:159541]

R989-X62-566: His6-MBP-tev-G-Hs.KRAS4b(1-169) Q61H

R988-X60-566: His6-MBP-tev-G-Hs.KRAS4b(1-169) T35S

R989-X11-566: His6-MBP-tev-G-Hs.KRAS4b(1-169) G13D [Addgene:159543]

R929-X18-566: His6-MBP-tev-G-Hs.KRAS4b(1-169) G13D/A130I

R929-X19-566: His6-MBP-tev-G-Hs.KRAS4b(1-169) G13D/A130L

R929-X20-566: His6-MBP-tev-G-Hs.KRAS4b(1-169) G13D/A130V

R929-X21-566: His6-MBP-tev-G-Hs.KRAS4b(1-169) G13D/A130F

R702-X66-566: His6-MBP-tev-Hs.RAF1(52-131).

### Protein expression and purification

RAF1(52-131) was expressed using the auto-induction media protocol, and KRAS proteins were expressed using the Dynamite media protocol^43^. Gly-Hs.KRAS4b(1-169) G13D was expressed following published protocols for ^15^N incorporation^44^. Gly-Hs.KRAS4b(1-169) G13D was expressed following published protocols for ^13^C/^15^N incorporation with modifications^45^. Specifically, ZnCl_2_ was omitted, and IPTG induction was done at either 16 °C or 20 °C to provide a higher yield. Highly deuterated, ^13^C-methyl/^15^N-labeled KRAS proteins were expressed as previously described^21,46^. All proteins were purified as previously outlined for Gly-Hs.KRAS (1-169)^47^; MgCl_2_ was omitted when purifying RAF1(52-131).

### NMR relaxation dispersion experiments

All relaxation dispersion experiments were carried out on a 700 MHz (16.4 T) Bruker Avance NEO spectrometer equipped with a helium-cooled TCI cryoprobe. All methyl relaxation dispersion experiments^25,48,49^ were performed using U-[^15^N, ^2^H], ^13^CH_3_-δ_1_-Ile-labeled KRAS^G13D^ samples (1 − 169) with a concentration of about 250 μM in a 20 mM HEPES (pH 7.4), 150 mM NaCl, 5 mM MgCl_2_, and 2 mM TCEP buffer. A γB_1_ frequency of ∼15 kHz was used for the ^13^C π pulse in a 20 ms constant-time spin-echo period of ^13^C single-quantum CPMG experiments, with νCPMG frequencies of 100, 200, 400, 500, 800, and 1000 Hz, with repetition of the 100, 400, and 1000 Hz data points. On the other hand, in methyl adiabatic relaxation dispersion experiments (HARD), only four different composite hyperbolic secant (HS) pulses were used to measure different relaxation rates in adiabatic R_1ρ_ (HS1, HS2, HS4, and HS8) and R_2ρ_ (HS1, HS4, HS8, and HS4_1ms) experiments. All the data were processed by NMRPipe and NMRFAM-Sparky^50^ before further analysis. During the final analysis, CPMG data and HARD data were fit with 2-site exact analytic solution and 2-site solution surface repectively. All the fits started with an extensive grid search and ended with an accelerated gradient descent by assuming the Arrhenius equation and a fixed Δω value at different temperatures.

Amide relaxation dispersion experiments were performed using U-[^15^N] labeled GMPPNP-bound- KRAS^G13D^(1 − 169) samples with a concentration of about 400 μM in the same buffer conditions. TROSY ^15^N single-quantum CPMG experiments^51^ were carried out with a 20 ms constant time, 2 s recycle delay, 32 scans, 128 complex points in the ^15^N dimension (35 ppm), and 2048 complex points in the ^1^H dimension (14 ppm) at 25 °C. A γB_1_ frequency of ∼7 kHz was used for the ^15^N π pulse in the constant-time spin-echo period, with νCPMG values of 100, 200, 400, 500, 800, and 1000 Hz, with repetition of the 100, 400, and 1000 Hz data points. All the data were processed with a linear prediction of an additional 128 complex points in the ^15^N dimension using NMRPipe^52^ and subsequently analyzed in NMRFAM-Sparky^50^. Experimental values of the relaxation dispersion intensities and analysis data are provided as a Supplementary Data 2.

### ^31^P NMR

1D-^31^P NMR spectra were acquired at 5 °C on a Bruker 500 MHz NMR spectrometer (202 MHz ^31^P frequency) with a 5 mm Prodigy broadband cryogenic probe using 70° flip angle pulses, 1200 scans, an interscan delay of 7 s, an acquisition time of 84 ms, and a WALTZ-16 proton decoupling sequence. The 1D-^31^P NMR experiments were repeated with similar conditions (except those of double-mutants), and the resulting spectra showed no visible difference from the original ones.

### Assignments

Resonance assignments of KRAS^G13D^, in both GDP and GMPPNP-bound- states, were obtained from the analysis of a standard set of two-dimensional (2D) and three-dimensional (3D) NMR spectra: 2D HN-HSQC, 3D HNCACB, 3D CBCA(CO)NH, and 3D C(CO)NH and deposited in BMRB (Entry: 51642). All 3D experiments were recorded using non-uniform sampling (NUS) at a sampling rate of 40%, and the spectra were reconstructed using the SMILE^53^ and NMRPipe^52^ software packages.

### Residual dipolar couplings

KRAS^G13D^ and KRAS^WT^ NMR samples contained 0.25–1.2 mM of uniformly [^15^N]- or [^13^C/^15^N]-labeled protein in GDP and GMPPNP-bound states in 20 mM HEPES pH 7.4, 150 mM NaCl, 2 mM MgCl_2_, 1 mM TCEP and 5% D_2_O in susceptibility matched 5 mm Shigemi microtubes. Anisotropic samples additionally contained 20 mg/ml Pf1 phage particles (Asla Biotech, Riga, Latvia), resulting in an HDO deuterium signal splitting of 13.1 Hz in lines with 1 Hz linewidths, or 6% w/v of q = 3.0 DMPC/DHPC bicelles (Avanti Polar Lipids, Alabaster, AL), resulting in an HDO deuterium signal splitting of 10.2 Hz in lines with 1 Hz linewidths. All RDC NMR spectra were acquired on a Bruker 700 MHz NMR spectrometer at 25 °C (except the bicelles RDC spectra, measured at 32 °C). ^1^D_NH_ and ^1^D_CaHa_ RDCs were measured using a 3D-HNCO variant of the ARTSY^54^ experiment and a 3D HCA(CO)N antiphase ^1^H-coupled in the ^13^C^α^ dimension experiment, respectively. The ^1^D_NC’_ and ^2^D_H_^N^_C’_ were measured from a 3D IPAP-J-HNCO pulse sequence included in the Bruker spectrometer pulse sequence library. All RDC experiments were acquired with sufficient S/N to ensure low experimental errors, as represented by the error bars in Supplementary Figs. 5, 6, and 7 (some become smaller than the dot size for experimental RDCs with large ranges of magnitude). Experimental values of all RDCs are provided as a Supplementary Data 1.

Since each RDC set measured in a particular alignment medium does not sample all possible spatial bond orientations in the alignment tensor frame (or, equivalently, in the molecular frame), the RDC-fitted magnitude of the corresponding alignment tensor using singular value decomposition (SVD) is often underestimated by about 10%. In contrast, the rhombicity of the tensor is unaffected. We performed a grid search that imposed slightly larger alignment tensor magnitudes to compensate for this ‘sparsity underestimate’ and determined the optimal magnitude of the alignment tensors for each alignment medium using all experimental RDCs measured in that medium and the crystal structure (State 1). These corrected alignment tensor magnitudes provided the best RDC fit to the State I crystal structure (lowest R factors^33^) for all types of RDCs measured in each alignment medium. The result was indeed between 7% and 12% larger than those obtained in a simple SVD fit of any individual set of RDCs. We subsequently fixed these optimal values of each alignment tensor throughout the structure calculations of the two-state ensemble.

### Structure calculations

We used Xplor-NIH^55^ to perform an *N* = 2 ensemble RDC refinement protocol in torsion angle coordinates, during which one ensemble member was fixed throughout to the crystal structure of State 1 (ground state). Since we were not refining the positions of the switch regions (due to the lack of NMR restraints) and the high-resolution crystal structures of State 1 and State 2 conformations are highly similar (RMSD of only 0.1–0.2 Å when the switch regions are excluded), either structural KRAS^G13D^ model could have been equally employed as a starting point of the *N* = 2 ensemble refinement. Consequently, the calculated GMPPNP-bound- KRAS^G13D^ minor state represents the intermediate state, while its switch regions remain close to their State 1 conformation. The refinement protocol utilized population-weighted RDC and Talos-N dihedral angle restraints from NMR experiments in addition to standard Xplor-NIH energy terms, using a script that allows up to 3 Å departures from the initial (crystal structure) atomic positions in a non-crystallographic symmetry type of potential. All 3D structural representations were made in PyMol^56^.

### Crystallization and structure determination of active KRAS^G13D^ and KRAS^G13D^-RAF1-RBD complex

To crystallize KRAS^G13D^ bound to GMPPNP, we first carried out nucleotide exchange to replace GDP with GMPPNP using the protocol described previously^57^. Crystallization screenings were carried out using the sitting-drop vapor diffusion method by mixing the protein (8 mg/ml) with an equal volume of reservoir solution. Initial crystals were obtained using a reservoir solution consisting of 100 mM Tris pH 8.5, 20% PEG 400, 20% PEG 8000, and 50 mM MgCl_2_. To improve crystal quality, microseeding was used to generate single 3D crystals from the initial rod clusters. Crystals were flash-frozen in liquid nitrogen without any additional cryo-protectant. A crystallization sample of KRAS^G13D^ bound to RAF1-RBD was prepared as described previously^58^, and crystals were obtained using the reservoir solution containing 0.15 M KBr and 30% PEG-MME2K. Diffraction data were collected on a 24-ID-E beamline at Advanced Photon Source (APS), Argonne National Laboratory (ANL). Data were integrated and scaled using XDS^59^. Structure solution was obtained with molecular replacement using Phaser as implemented in the Phenix programs suite, using the GMPPNP-bound KRAS^Q61H^ mutant (PDB: 3GFT, the best GMPPNP-bound KRAS model available at the time) as the search model for KRAS^G13D^ bound to GMPPNP and KRAS-RAF1(RBD) complex (PDB: 6VJJ) as a search model for the structure of KRAS^G13D^ bound to RAF1-RBD^60^. Iterative model building and refinement were performed with COOT^61^ and Phenix.Refine^62^. Crystal parameters, data collection statistics, and refinement statistics are summarized in Table 1. The SBGrid consortium^63^ provided crystallographic and structural analysis software support.

### ITC measurements

A MicroCal PEAQ-ITC calorimeter (Malvern) was used to perform ITC binding studies. GMPPNP-bound KRAS and RAF1-RBD used in ITC measurements were extensively dialyzed in a buffer containing 150 mM NaCl or 300 mM NaCl with 20 mM HEPES (pH 7.3), 5 mM MgCl_2_, and 1 mM TCEP. The concentrations of the proteins were measured using absorbance at 280 nm in a NanoDrop 2000c spectrophotometer (Thermo Fisher Scientific). Before each ITC run, protein samples were centrifuged at 14,000 g for 5 min at 4 °C to remove debris and air bubbles. ITC titrations were performed at 25 °C by an initial injection of 0.4 μL followed by 18 injections of 2.2 μL of RAF1-RBD proteins at 150 s intervals into a cell containing KRAS mutant proteins. Data analysis was performed using a “one set of sites” model using the MicroCal PEAQ-ITC analysis software (v1.41, Malvern Panalytical) to obtain thermodynamic parameters (ΔG, ΔH, and −TΔS), curve fitting, equilibrium dissociation constant, and molar ratio calculations. All binding and thermodynamic data obtained from ITC experiments (*n* = 1) are tabulated in Supplementary Table 2. Errors in the *K*_D_ and ΔH values correspond to the standard error of fitting. The ITC raw data are provided as Supplementary Data 3 and 4.

### Statistics and reproducibility

All the NMR data, X-ray crystallography data, and ITC data were carried out as single measurements (no replicates).

### Reporting summary

Further information on research design is available in the Nature Portfolio Reporting Summary linked to this article.

## Supplementary information

Cornilescu_Peer review file

Supplementary Information

Description of Additional Supplementary Files

Supplementary Data 1

Supplementary Data 2

Supplementary Data 3

Supplementary Data 4

Reporting Summary

## Data Availability

GMPPNP-bound KRAS^G13D^ assignments were performed using NMRFAM-Sparky^50^ and were deposited in BMRB (Entry: 51642). The atomic coordinates and structure factors of the GMPPNP-bound KRAS^G13D^ and KRAS^G13D^ in complex with RAF1-RBD were deposited in the Protein Data Bank with accession codes 8EBZ and 8EPW, respectively. Experimental values of all RDCs, relaxation dispersion intensities and analysis data, and ITC raw data are provided as Supplemental Excel spreadsheets. All other data are available from the corresponding authors on reasonable request.
