# Supplementary material for: Reduced dynamic complexity allows structure elucidation of an excited state of KRASG13D
Source: Commun Biol. 2023 Jun 2;6:594. doi: 10.1038/s42003-023-04960-6 (PMC10238545; doi:10.1038/s42003-023-04960-6)
Supplement: Supplementary file 2 — Supplementary Information [file 42003_2023_4960_MOESM2_ESM.pdf]

Supplementary Information

Identification and Structure Elucidation of an Excited State in a KRAS G13D Oncogenic Mutant with Reduced Dynamic Complexity

Fa-An Chao[1]*, Albert H. Chan[1], Srisathiyanarayanan Dharmaiah[1], Charles D. Schwieters[2], Timothy H. Tran[1], Troy Taylor[1], Nitya Ramakrishnan[1], Dominic Esposito[1], Dwight V. Nissley[1], Frank McCormick[1,3], Dhirendra K. Simanshu[1]*, Gabriel Cornilescu[1]*

[1]NCI RAS Initiative, Cancer Research Technology Program, Frederick National Laboratory for Cancer Research, Leidos Biomedical Research, Frederick, Maryland 21701, USA.

[2]Division of Computational Bioscience, Center for Information Technology, National Institutes of Health, Building 12A, 20892-5624, Bethesda, MD, USA.

[3]Helen Diller Family Comprehensive Cancer Center, University of California San Francisco, 1450 3rd Street, San Francisco, California 94158, USA.

Correspondence: fa-an.chao@nih.gov (F-A.C.), dhirendra.simanshu@nih.gov (D.K.S), gabriel.cornilescu@nih.gov (G.C.)

This PDF file includes:

Supplementary Figures 1–11

Supplementary Tables 1–2

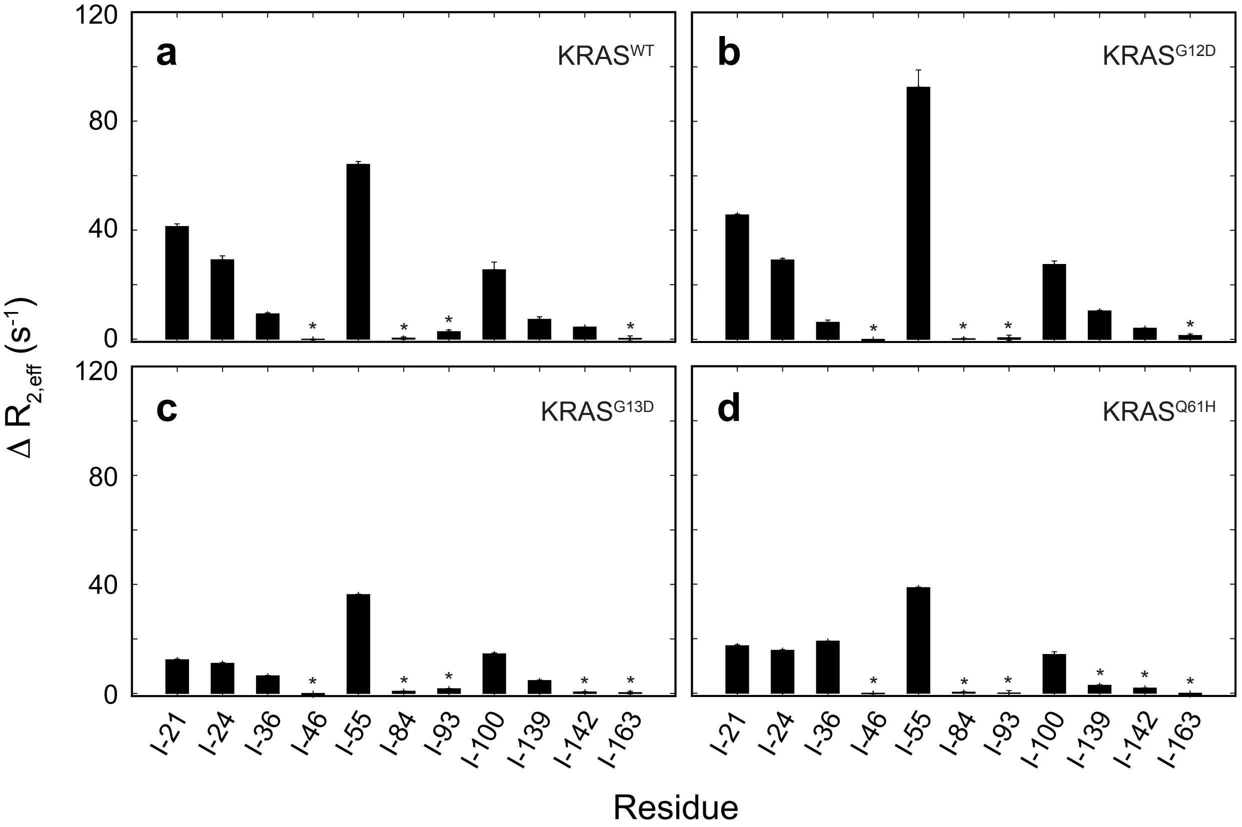

Supplementary Figure 1. Amplitudes of methyl relaxation dispersion profiles.
Amplitudes of methyl relaxation dispersion profiles of δ_1-labeled methyl groups of isoleucine residues in GMPPNP-bound KRAS^{WT} (**a**) and GMPPNP-bound KRAS mutants (**b, c, d**) at 25 °C. A γB_1 frequency of ~15 kHz was used for the ^{13}C π pulse in the constant-time spin-echo period, with νCPMG values of 100, 200, 400, 500, 800, and 1000 Hz, with repetition of the 100, 400, and 1000 Hz data points. The amplitudes were calculated as the differences between effective R_2 rates at 100 and 1000 Hz. The measurement errors were estimated by the replicates at three νCPMG values in the profiles. The bars represent a single datapoint and the error represents the propagated NMR peak amplitude measurement error in each fitted relaxation dispersion curve. The experimental errors were previously estimated as 1 s^{-1} (16), therefore resonances marked by asterisks having amplitudes less than 3 s^{-1} were not included in the analysis.

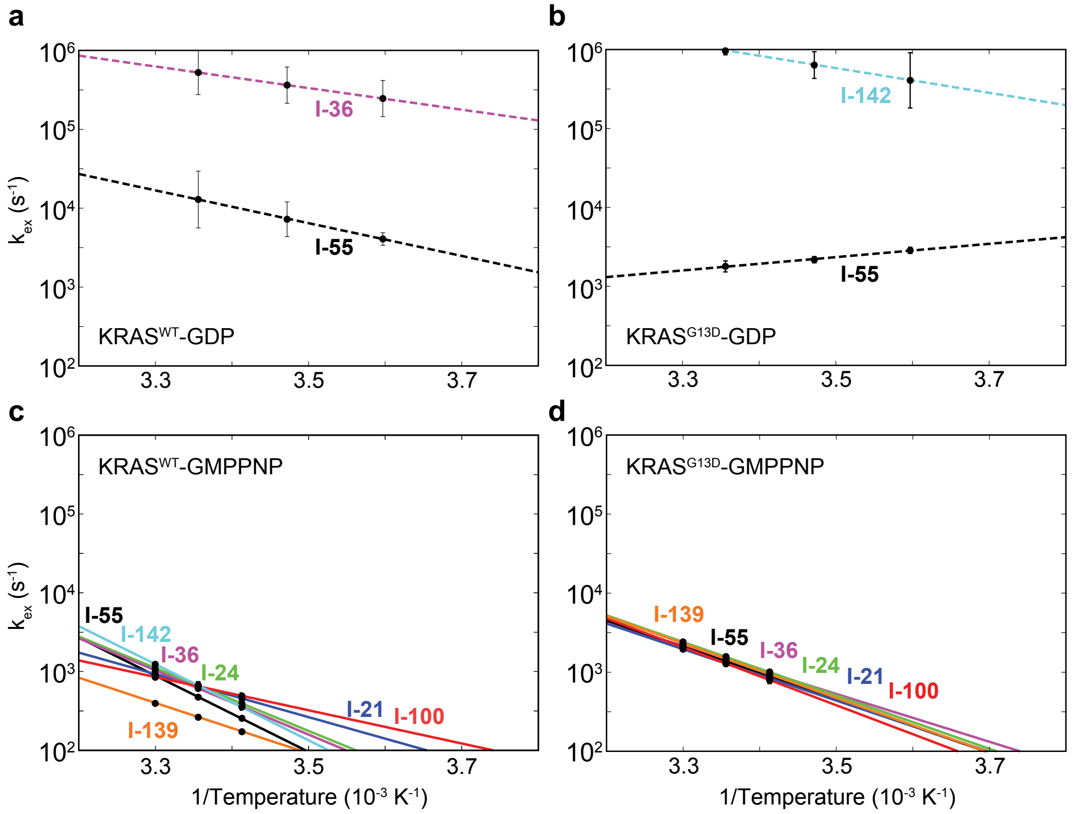

Supplementary Figure 2. Temperature-dependent conformational dynamics of KRAS^{WT} and KRAS^{G13D} studied by δ_1-labeled methyl groups of isoleucine residues.

The data of GDP-bound KRAS, GDP-bound KRAS^{G13D}, GMPPNP-bound KRAS, and GMPPNP-bound KRAS^{G13D} are plotted in **a**, **b**, **c**, and **d**, respectively. Each methyl group data set is fitted separately, assuming a simple two-site exchange model with reaction rates (k_1 and k_{-1}) following the Arrhenius equation and a fixed chemical shift difference ($\Delta\omega$) at three different temperatures. The calculated exchange rates ($k_{ex} = k_1 + k_{-1}$) are plotted against temperatures. The errors of the fitting are reported as the standard deviations among the 10 best-fitting results out of 100 that start from randomly selected initial points. The solid-line (GMPPNP-bound) and dash-line (GDP-bound) data for KRAS proteins were generated from SQ-CPMG experiments and adiabatic relaxation dispersion experiments, respectively. Since little or no relaxation dispersion is observed for GDP-bound KRAS in CPMG experiments, adiabatic relaxation dispersion experiments were used to probe the faster exchange regime. As described previously (16), only methyl groups with detectable conformational dynamics are reported. Residues I-21, I-24, I-36, I-55, I100, I-139, and I-142 are shown in blue, green, purple, black, red, orange, and cyan, respectively.

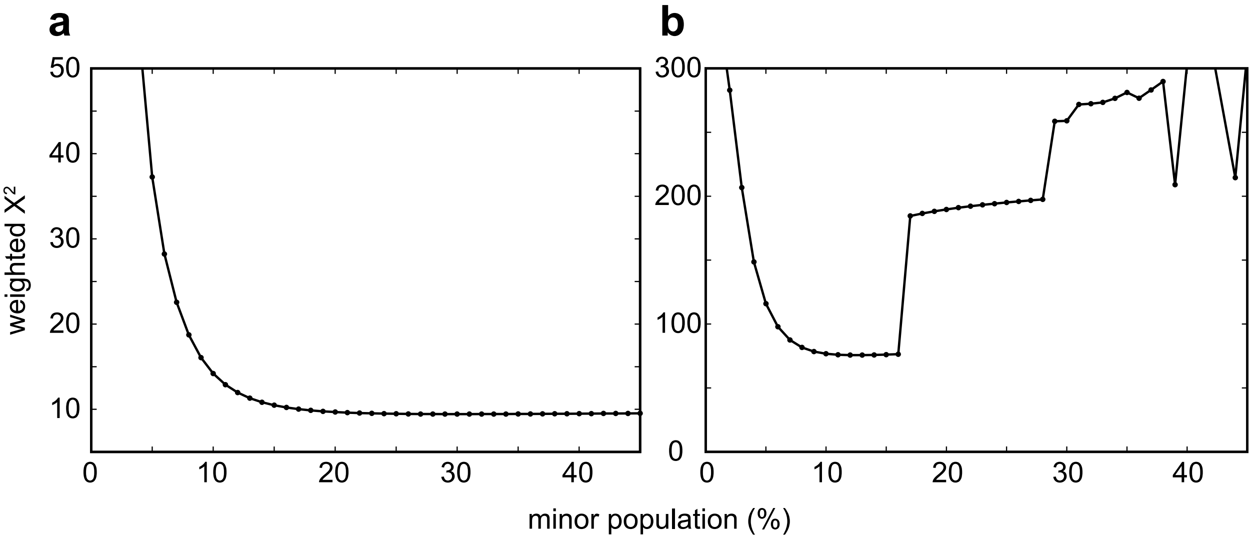

Supplementary Figure 3. **χ^2 surfaces of global data analysis of methyl relaxation dispersion experiments on GMPPNP-bound KRAS^{G13D}**.

a Assuming the simple two-site exchange, the χ^2 surface of global data analysis of all methyl groups with detectable dispersion profiles at 25 °C is plotted against the minor population. **b** Assuming the simple two-site exchange and Arrhenius equation, the χ^2 surface of global data analysis of all methyl groups with detectable dispersion profiles at three different temperatures is plotted against the minor population.

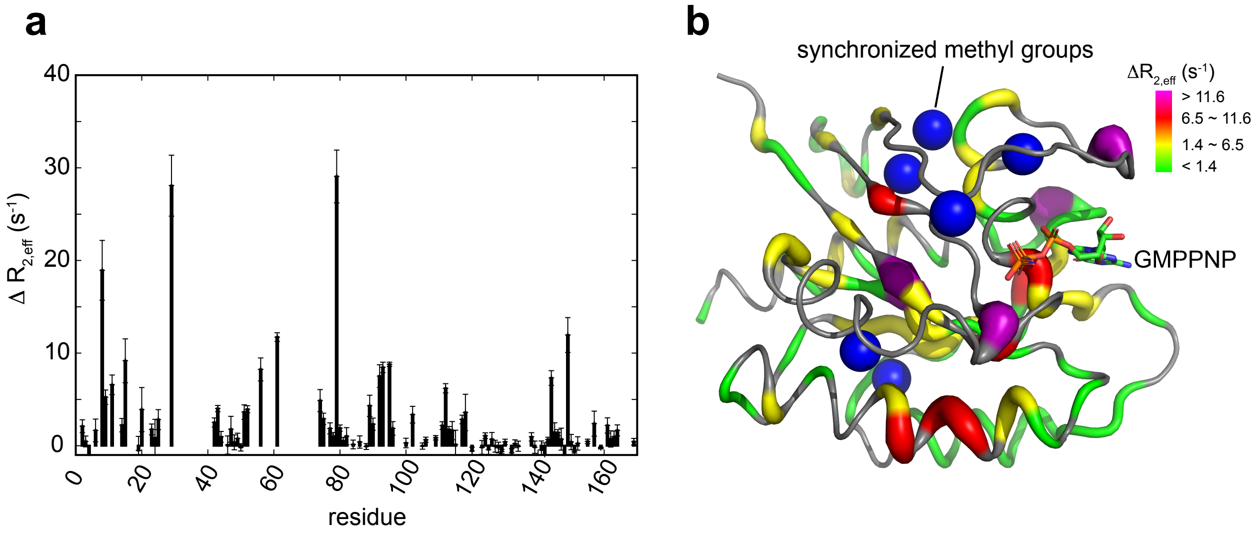

Supplementary Figure 4. Conformational dynamics revealed by amide relaxation dispersion experiments in GMPPNP-bound KRAS^{G13D}.

a Amplitudes of relaxation dispersion profiles from TROSY-version ^{15}N single-quantum CPMG with a 20 ms constant time are plotted against residue number. The amplitudes are calculated using differences between $R_{2,eff}$ values at νCPMG frequencies of 100 and 1000 Hz. The measurement errors were estimated by the replicates at three different νCPMG values in the profiles. The bars represent a single datapoint and the error represents the propagated NMR peak amplitude measurement error in each fitted relaxation dispersion curve. **b** Amplitudes of relaxation dispersion profiles are mapped onto the crystal structure (PDB: 8EBZ), and residues with no data are colored gray. The methyl groups with the detectable synchronized motion are marked as blue spheres.

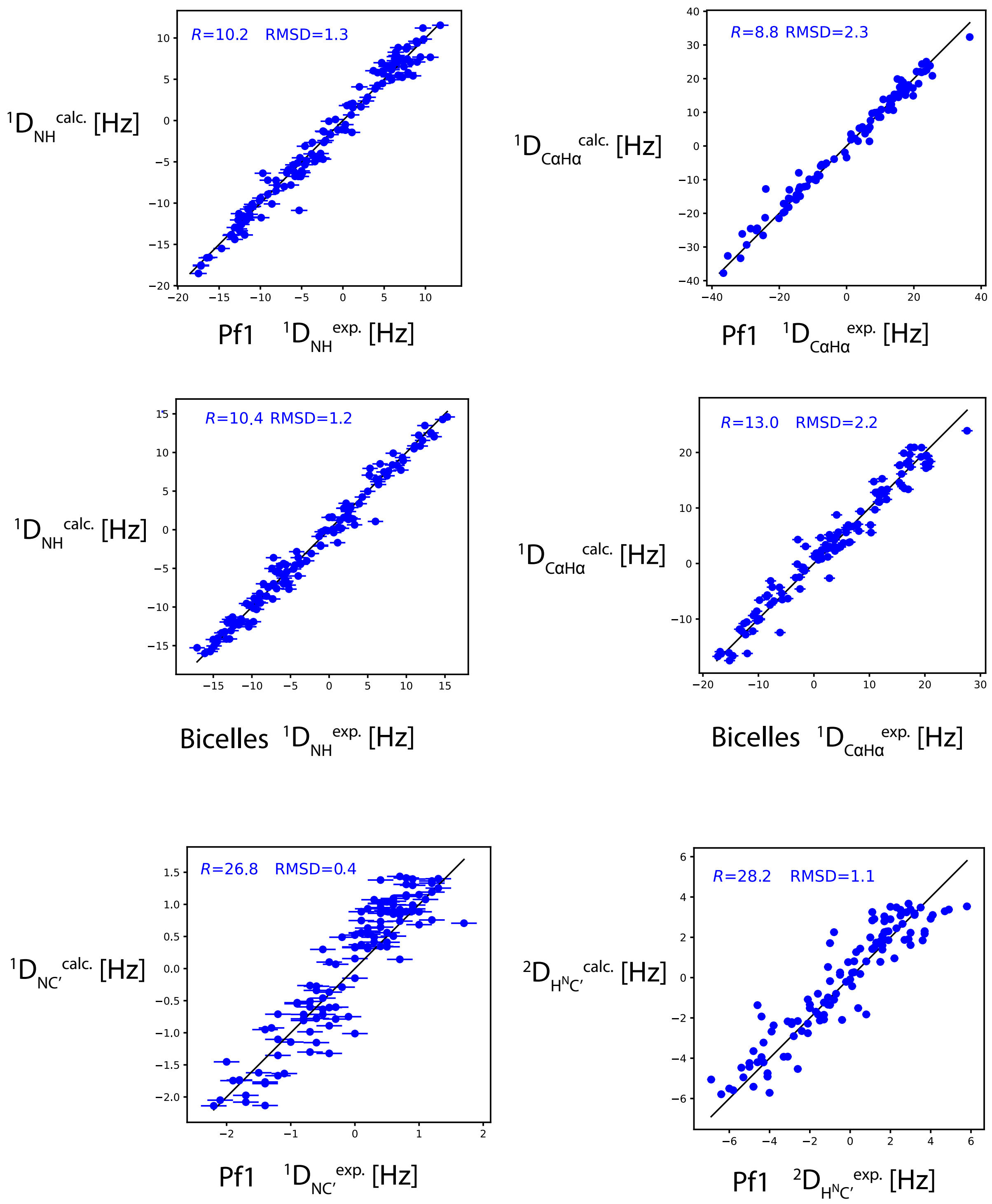

Supplementary Figure 5. RDCs are not over-fitted.

The RMSD (in Hz) of the RDC fit to the N=2 ensemble of calculated KRAS-G13D structures matches their corresponding measurement precision.

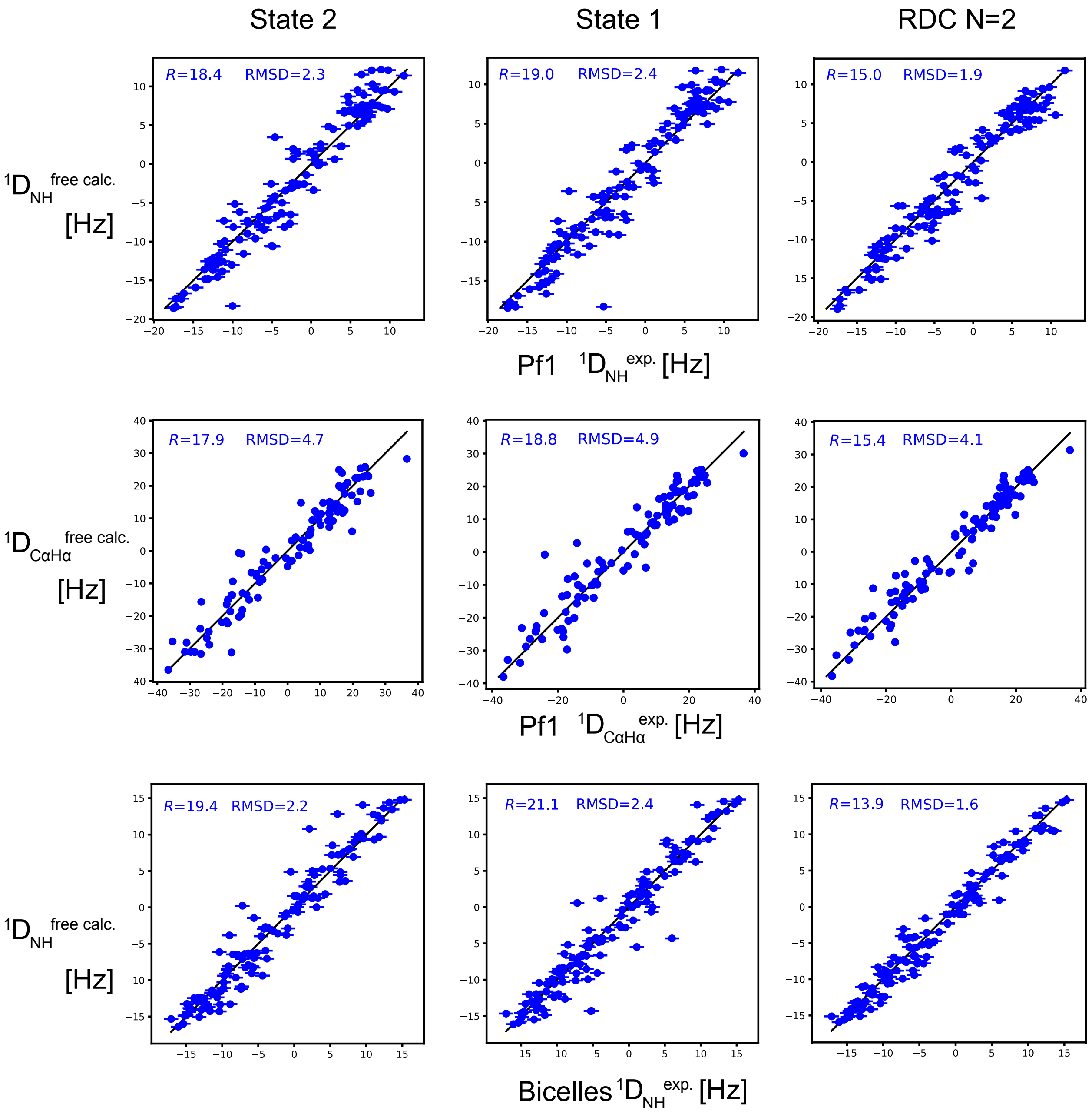

(continued on next page)

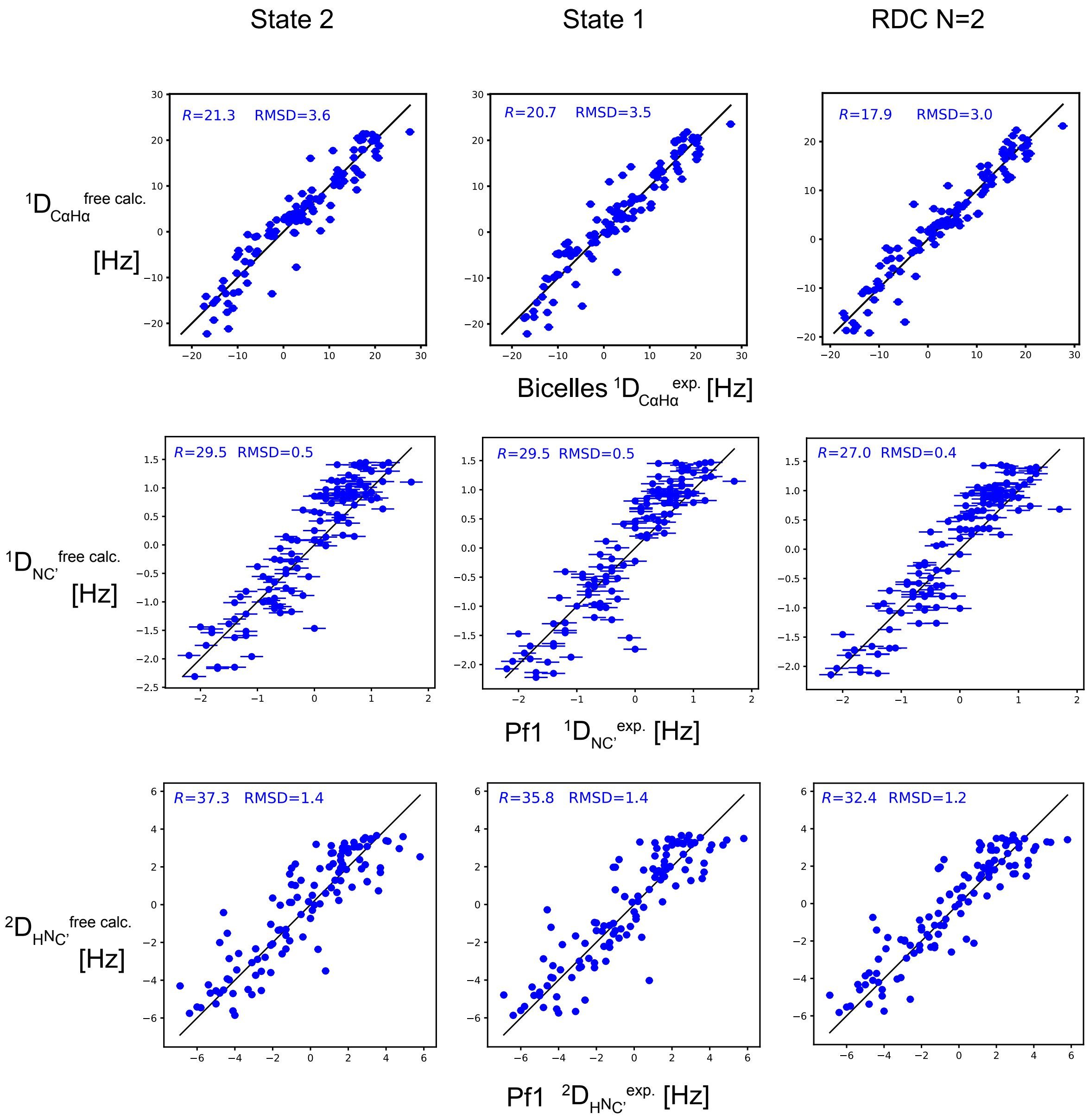

Supplementary Figure 6. Structural cross-validation of the GMPPNP-bound KRAS^{G13D} minor state using individual sets of RDCs systematically excluded from the RDC-refined N=2 ensemble calculation.

Each RDC set was excluded from the RDC-refined N=2 ensemble calculation and fitted to the resulting structures. The N=2 panels in each row show consistent improvements in the R=R^{free} values of the RDC-refined N=2 ensemble compared to the fit to the representative crystal structures for States 2 and 1 (R values).

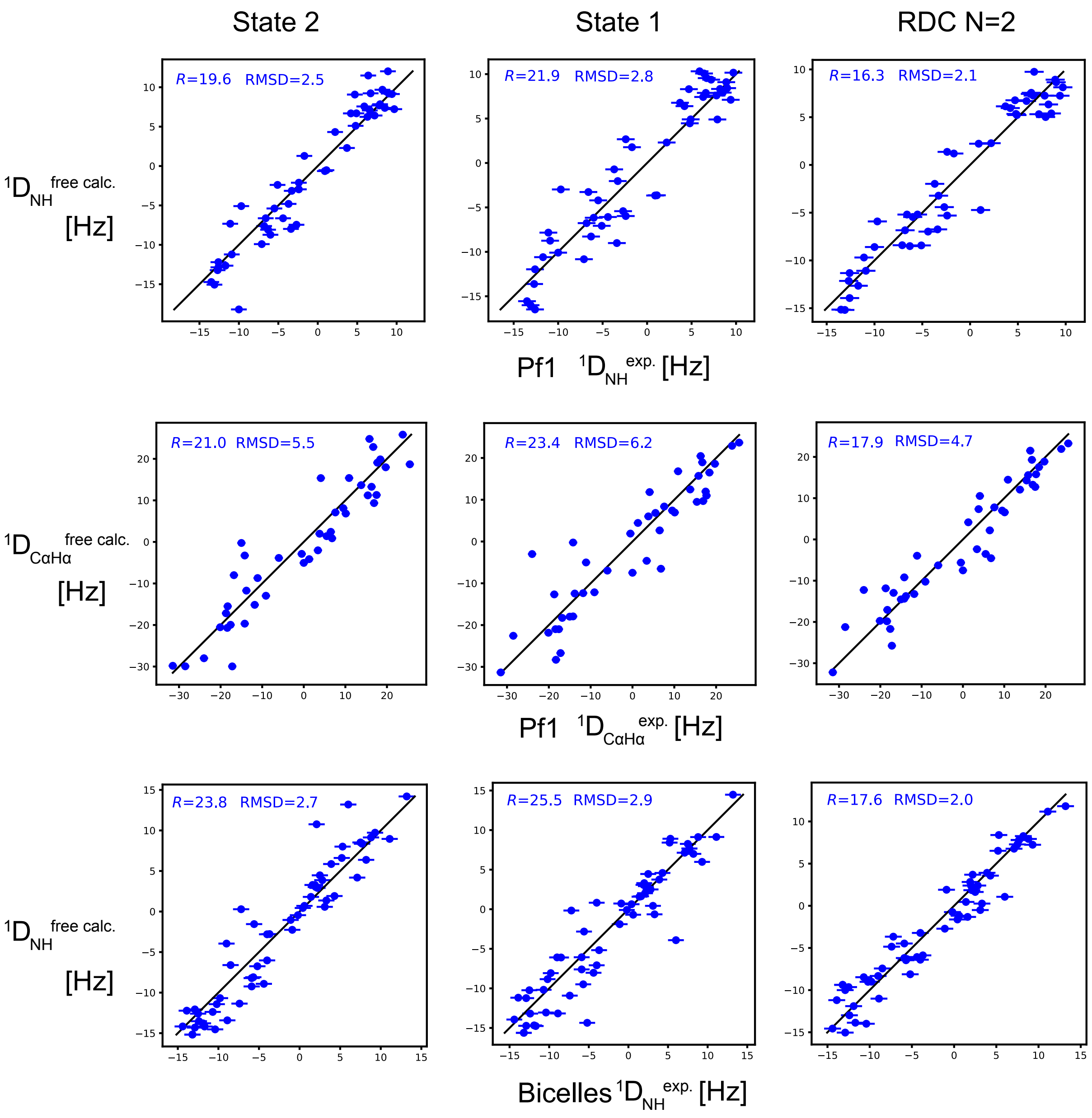

(continued on next page)

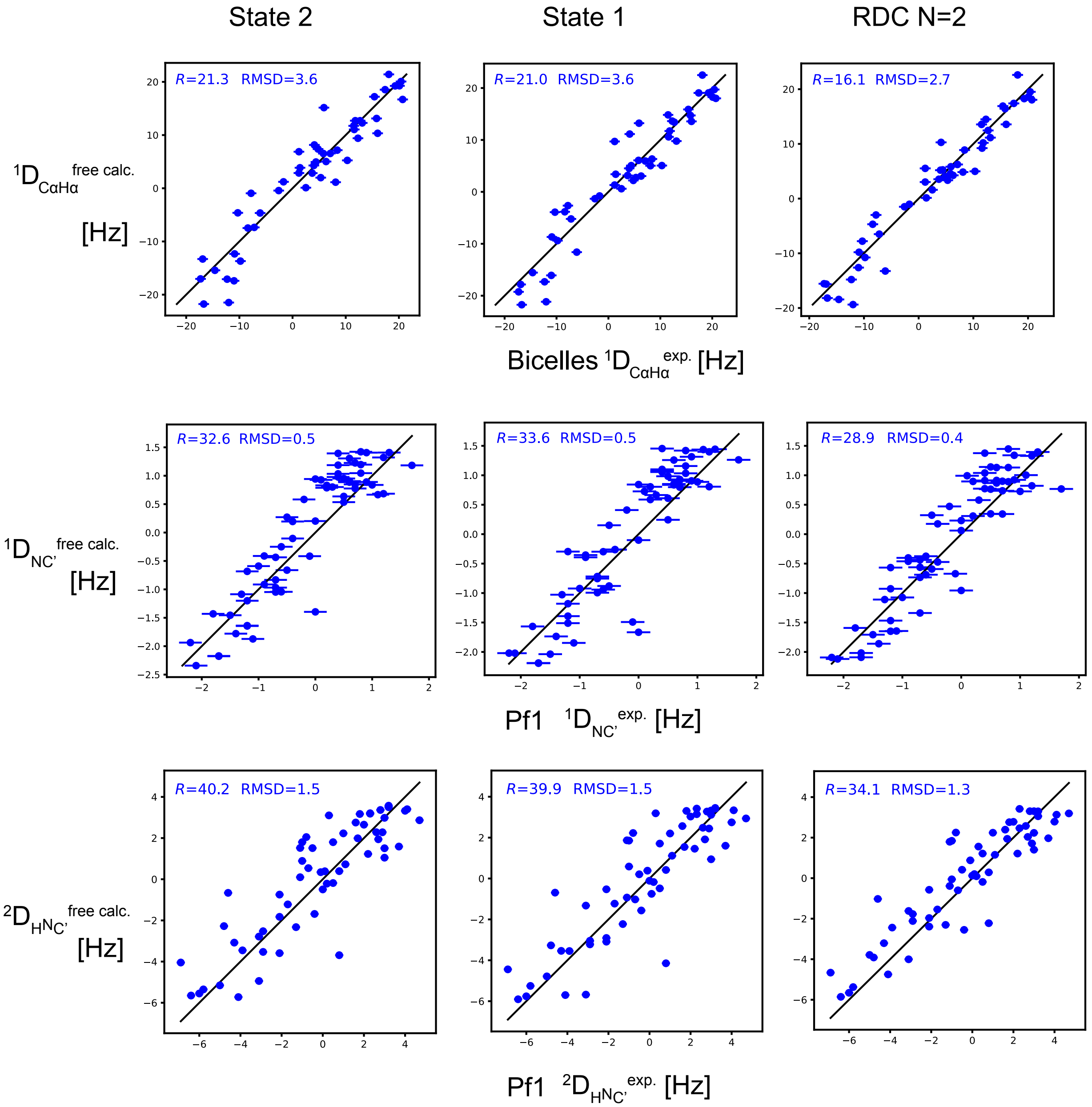

Supplementary Figure 7. Structural cross-validation of the GMPPNP-bound KRAS^{G13D} minor state when selecting only the residues showing the most significant changes in the spatial orientation of the RDC vectors.

Each RDC set was excluded from the RDC-refined N=2 ensemble calculation and fitted to the resulting structures. The N=2 panels in each row show consistent improvements in the R=R^{free} values of the RDC-refined N=2 ensemble compared to the fit to the representative crystal structures for States 2 and 1 (R values). RDCs of residues 7, 13-15, 23-29, 43-54, 60-63, 73-81, 86, 92-95, 105-113, 120-124, 137-140, 144-150 are shown.

a

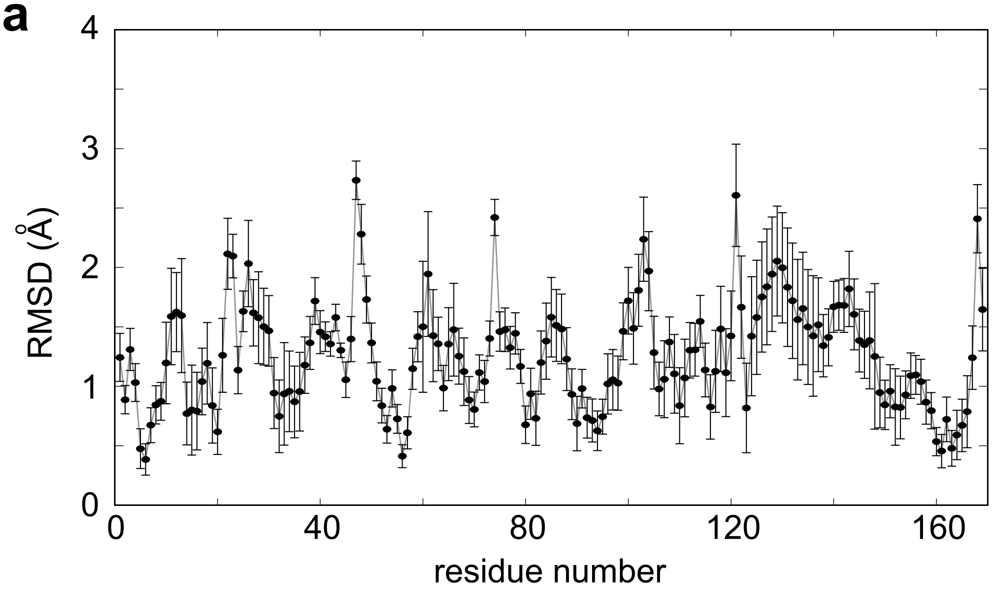

b

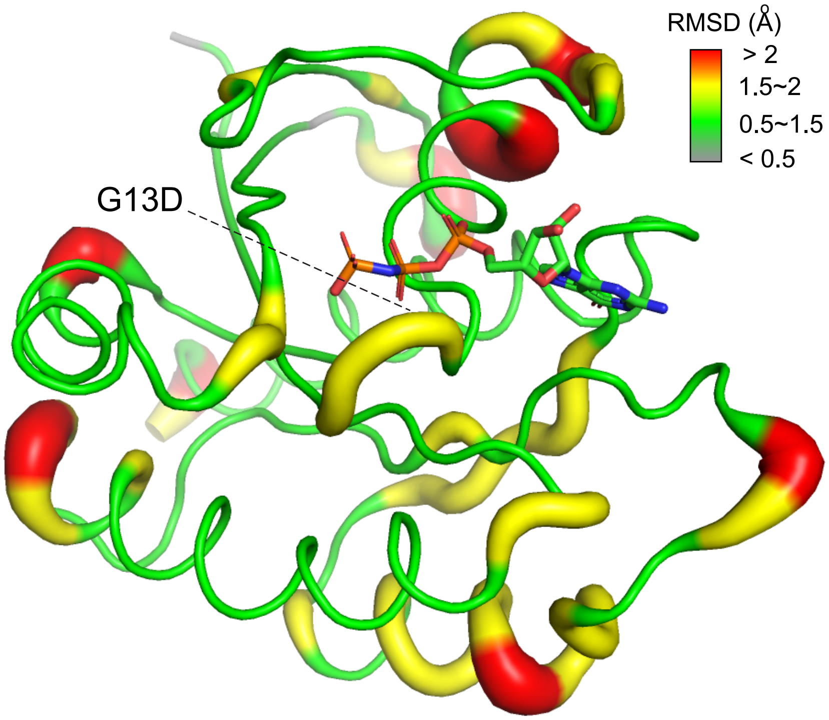

Supplementary Figure 8. **The average RMSD between the ground-state structure and the excited-state structural ensemble of KRAS^{G13D}.**

a The average RMSD and the standard deviation of each residue are plotted against the primary sequence. The standard deviation is calculated based on each RMSD of 10 conformers of the excited state against the ground-state structure. **b** The average RMSD values are mapped onto the crystal structure of KRAS^{G13D} (PDB: 8EBZ).

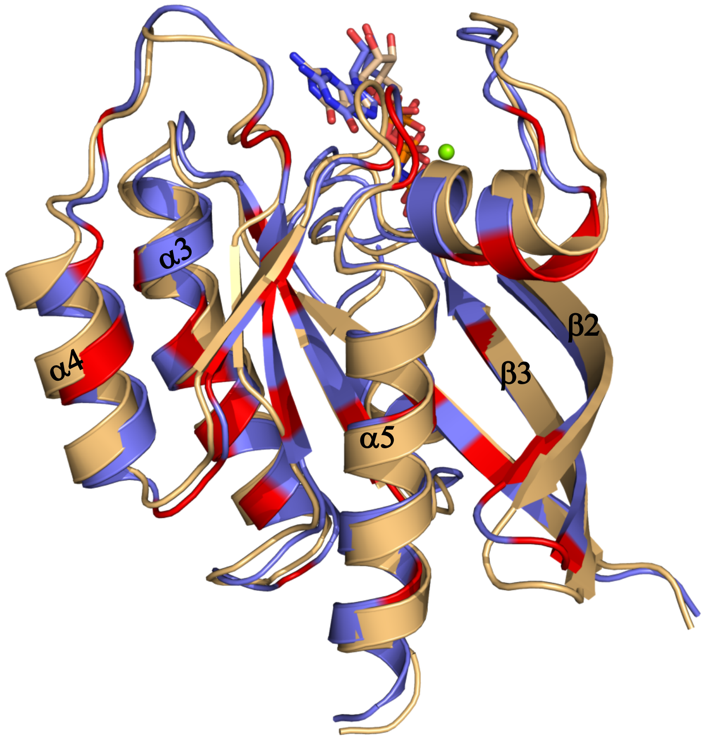

Supplementary Figure 9. **Overlay of the major and minor state models of KRAS^{G13D}.** The residues having an experimental RDC difference between KRAS^{G13D} and KRAS^{WT} larger than 1.5 times the overall RDC RMSD between the two species are highlighted in red on the KRAS^{G13D} State 1 crystal structure (major conformer, in blue). The minor conformer (RDC) is shown in gold.

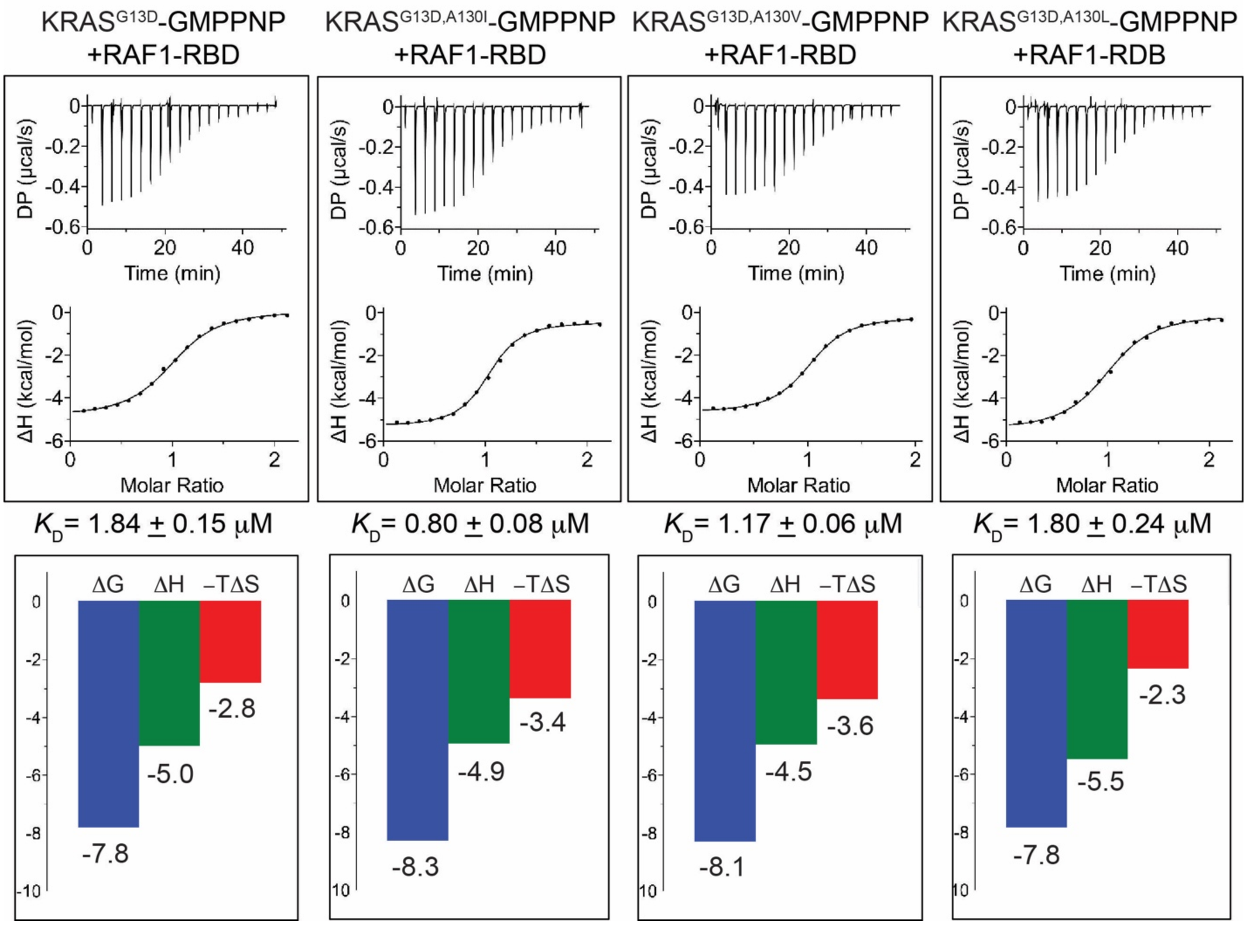

Supplementary Figure 10. The RAF1-RBD binding affinities of KRAS mutations in the intermediate state cavity.

Measurement by ITC at a high salt concentration (300 mM NaCl) and a low protein concentration (35μM KRAS) was performed in singlicate.

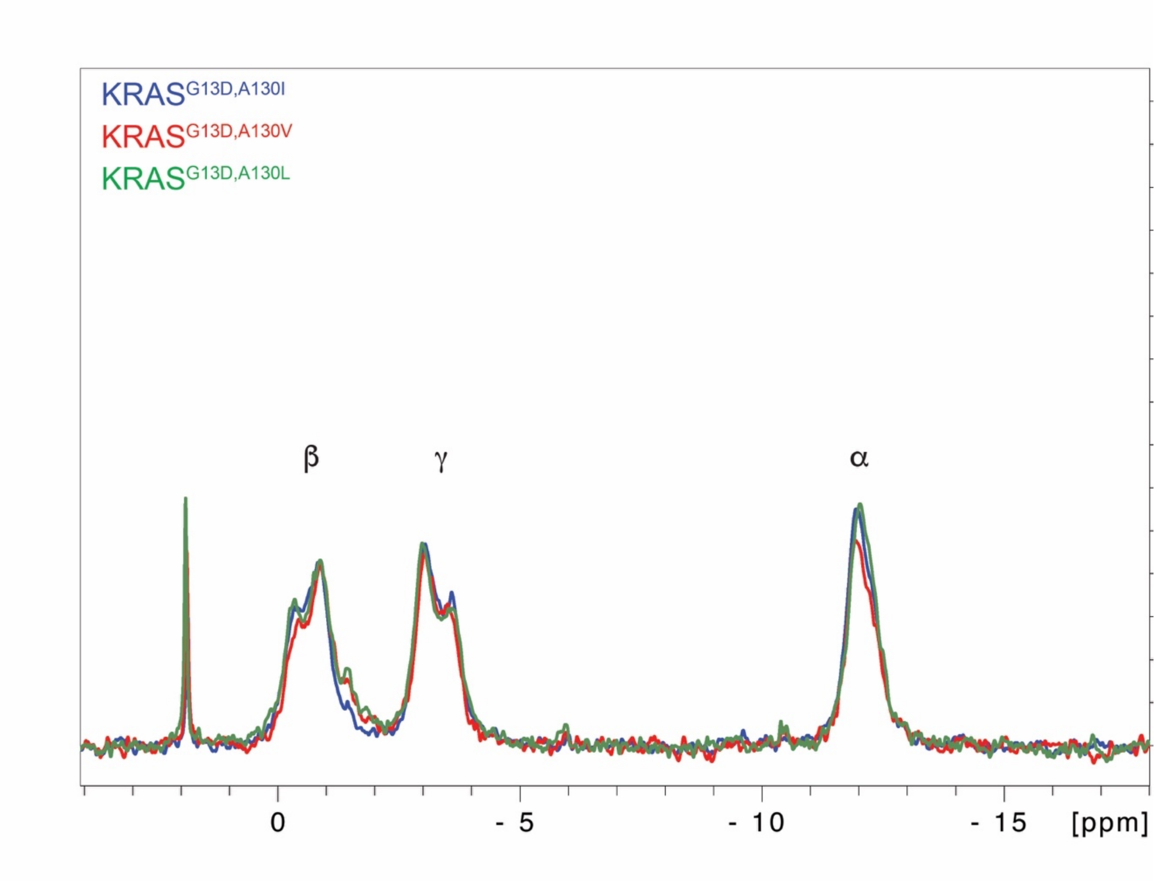

Supplementary Figure 11. KRAS^{G13D,A130V} and KRAS^{G13D,A130L} have almost identical population ratios to KRAS^{G13D,A130I}

Overlay of ^{31}P NMR spectra of KRAS^{G13D,A130I}, KRAS^{G13D,A130V}, and KRAS^{G13D,V130L}, all in GMPPNP-bound form, at 5 °C.

Supplementary Table 1. Cross-validation of the GMPPNP-bound $KRAS^{WT}$ minor state by systematically excluding individual sets of RDCs from the N=2 ensemble calculation.

The R^{free} factors show no consistent improvement over fitting the crystal structure representative of ground State 2.

**RDC**	**State 2 crystal**	**RDC N=2**
Pf1 $^1D_{NH}$	R = 24.9	R^{free} = 22.2
Pf1 $^1D_{C\alpha H\alpha}$	R = 17.6	R^{free} = 18.8
Bicelle $^1D_{NH}$	R = 22.9	R^{free} = 21.5
Bicelle $^1D_{C\alpha H\alpha}$	R = 22.4	R^{free} = 24.4

Supplementary Table 2. The thermodynamic parameters from ITC experiments measuring binding affinity of GMPPNP-bound KRAS mutants to RAF1-RBD.

**KRAS mutants (GMPPNP)**	**Binding Affinity (K_D) μM**	**ΔG (kcal/mol)**	**ΔH (kcal/mol)**	**-TΔS (kcal/mol)**	**N-Value**
Buffer: 20 mM HEPES pH-7.4, 150 mM NaCl, 5 mM $MgCl_2$, 1 mM TCEP<br>KRAS: 75 μM and RAF1-RBD: 750 μM					
W.T.	0.17 ± 0.04	-9.24	- 3.53 ± 0.06	-5.71	1.09
G13D	0.51 ± 0.05	-8.59	- 6.38 ± 0.05	-2.58	0.95
G13D, A130I	0.26 ± 0.02	-8.97	- 5.94 ± 0.04	-3.03	1.04
Buffer: 20 mM HEPES pH 7.4, 300 mM NaCl, 5 mM $MgCl_2$, 1 mM TCEP<br>KRAS: 35 μM and RAF1-RBD: 350 μM					
G13D	1.84 ± 0.15	-7.83	- 4.94 ± 0.08	-2.82	0.98
G13D, A130I	0.80 ± 0.08	-8.31	- 4.98 ± 0.06	-3.38	1.02
G13D, A130V	1.17 ± 0.06	-8.09	- 4.53 ± 0.04	-3.56	1.08
G13D, A130L	1.80 ± 0.24	-7.84	- 5.48 ± 0.14	-2.36	1.0

Thermodynamics parameters were calculated from a single ITC experiment (n=1) with 19-data points. Errors in the K_D and ΔH values correspond to the standard error of fitting. Thermodynamics parameters for $KRAS^{WT}$ from our earlier work[1] has been included here for comparison.

1. Chao FA, *et al.* Insights into the Cross Talk between Effector and Allosteric Lobes of KRAS from Methyl Conformational Dynamics. *J Am Chem Soc* **144**, 4196-4205 (2022).
