# Supplementary material for: Reduced dynamic complexity allows structure elucidation of an excited state of KRASG13D
Source: Commun Biol. 2023 Jun 2;6:594. doi: 10.1038/s42003-023-04960-6 (PMC10238545; doi:10.1038/s42003-023-04960-6)
Supplement: Supplementary file 3 — Description of Additional Supplementary Files [file 42003_2023_4960_MOESM3_ESM.pdf]

Description of Additional Supplementary Files

File name: Supplementary Data 1

Description: Experimental values of all RDCs used in structural refinement and validation.

File name: Supplementary Data 2

Description: The resonance intensities for each KRAS methyl group in methyl SQ-CPMG experiments at different temperatures and their dynamics analysis results. (Fig. 1b-e).

File name: Supplementary Data 3

Description: Source data for the ITC measurements at 150 mM NaCl (Fig. 6d).

File name: Supplementary Data 4

Description: Source data for the ITC measurements at 300 mM NaCl (Suppl. Fig. 10).
