# Supplementary material for: Reduced dynamic complexity allows structure elucidation of an excited state of KRASG13D
Source: Commun Biol. 2023 Jun 2;6:594. doi: 10.1038/s42003-023-04960-6 (PMC10238545; doi:10.1038/s42003-023-04960-6)
Supplement: Supplementary file 8 — Reporting Summary [file 42003_2023_4960_MOESM8_ESM.pdf]

nature portfolio

Corresponding author(s): Fa-An Chao, Dhirendra K. Simanshu, Gabriel Cornilescu

Last updated by author(s): 11 May, 2023

# Reporting Summary

Nature Portfolio wishes to improve the reproducibility of the work that we publish. This form provides structure for consistency and transparency in reporting. For further information on Nature Portfolio policies, see our Editorial Policies and the Editorial Policy Checklist.

## Statistics

For all statistical analyses, confirm that the following items are present in the figure legend, table legend, main text, or Methods section.

| n/a | Confirmed | |
|---|---|---|
| ☐ | ☒ | The exact sample size (*n*) for each experimental group/condition, given as a discrete number and unit of measurement |
| ☐ | ☒ | A statement on whether measurements were taken from distinct samples or whether the same sample was measured repeatedly |
| ☐ | ☒ | The statistical test(s) used AND whether they are one- or two-sided<br>*Only common tests should be described solely by name; describe more complex techniques in the Methods section.* |
| ☒ | ☐ | A description of all covariates tested |
| ☒ | ☐ | A description of any assumptions or corrections, such as tests of normality and adjustment for multiple comparisons |
| ☐ | ☒ | A full description of the statistical parameters including central tendency (e.g. means) or other basic estimates (e.g. regression coefficient) AND variation (e.g. standard deviation) or associated estimates of uncertainty (e.g. confidence intervals) |
| ☒ | ☐ | For null hypothesis testing, the test statistic (e.g. *F*, *t*, *r*) with confidence intervals, effect sizes, degrees of freedom and *P* value noted<br>*Give P values as exact values whenever suitable.* |
| ☒ | ☐ | For Bayesian analysis, information on the choice of priors and Markov chain Monte Carlo settings |
| ☒ | ☐ | For hierarchical and complex designs, identification of the appropriate level for tests and full reporting of outcomes |
| ☐ | ☒ | Estimates of effect sizes (e.g. Cohen's *d*, Pearson's *r*), indicating how they were calculated |

*Our web collection on statistics for biologists contains articles on many of the points above.*

## Software and code

Policy information about availability of computer code

| | |
|---|---|
| Data collection | NMR spectra were acquired using the Topspin (Bruker) program. |
| Data analysis | NMR spectra were reconstructed using the SMILE and NMRPipe software packages. Backbone dihedral angle restraints obtained from NMR chemical shifts using the Talos-N program. Crystallographic data were integrated and scaled using XDS. Structure solution was obtained with molecular replacement using Phaser as implemented in the Phenix programs suite. Iterative model building and refinement were performed with COOT52 and Phenix.Refine. The SBGrid consortium provided crystallographic and structural analysis software support. All 3D structural representations were made in PyMol. NMR ensemble calculation was carried out by XPLOR-NIH, and relaxation dispersion data were analyzed by previously reported in-house python scripts (10.1021/jacs.2c00007). |

For manuscripts utilizing custom algorithms or software that are central to the research but not yet described in published literature, software must be made available to editors and reviewers. We strongly encourage code deposition in a community repository (e.g. GitHub). See the Nature Portfolio guidelines for submitting code & software for further information.

# Data

Policy information about availability of data

All manuscripts must include a data availability statement. This statement should provide the following information, where applicable:

- Accession codes, unique identifiers, or web links for publicly available datasets
- A description of any restrictions on data availability
- For clinical datasets or third party data, please ensure that the statement adheres to our policy

GMPPNP-bound KRASG13D assignments were deposited in BMRB (Entry: 51642). The atomic coordinates and structure factors of the GMPPNP-bound KRASG13D and KRASG13D in complex with RAF1-RBD were deposited in the Protein Data Bank with accession codes 8EBZ and 8EPW, respectively.

# Human research participants

Policy information about studies involving human research participants and Sex and Gender in Research.

| | |
|---|---|
| Reporting on sex and gender | N/A. |
| Population characteristics | N/A. |
| Recruitment | N/A. |
| Ethics oversight | N/A. |

Note that full information on the approval of the study protocol must also be provided in the manuscript.

# Field-specific reporting

Please select the one below that is the best fit for your research. If you are not sure, read the appropriate sections before making your selection.

☒ Life sciences ☐ Behavioural & social sciences ☐ Ecological, evolutionary & environmental sciences

For a reference copy of the document with all sections, see nature.com/documents/nr-reporting-summary-flat.pdf

# Life sciences study design

All studies must disclose on these points even when the disclosure is negative.

| | |
|---|---|
| Sample size | N/A. |
| Data exclusions | The structural ensemble of the intermediate state in GMPPNP-bound KRASG13D was cross-validated by the improvement in the fit of each RDC set when iteratively excluded from the structural calculation restraints. |
| Replication | The 1D-31P NMR experiments were repeated with similar conditions (except those of double-mutants), and the resulting spectra showed no visible difference from the original ones. |
| Randomization | Each methyl group data set contains three measurements at three different temperatures (30, 25, and 20 C) that are fitted separately, assuming a simple two-site exchange model following the Arrhenius equation and a fixed chemical shift difference at different temperatures. The errors of the fits are reported as the standard deviations among the 10 best fits out of 100 fits. Each fit starts from randomly selected initial points, uses extensive grid search with simulating annealing, and then applies accelerated gradient descent. The methyl data are reported only when the amplitudes of their relaxation dispersion profiles at 25 C are well above the experimental errors (1 s-1). |
| Blinding | N/A. |

# Reporting for specific materials, systems and methods

We require information from authors about some types of materials, experimental systems and methods used in many studies. Here, indicate whether each material, system or method listed is relevant to your study. If you are not sure if a list item applies to your research, read the appropriate section before selecting a response.

## Materials & experimental systems

| n/a | Involved in the study |
| --- | --- |
| ☒ | ☐ Antibodies |
| ☒ | ☐ Eukaryotic cell lines |
| ☒ | ☐ Palaeontology and archaeology |
| ☒ | ☐ Animals and other organisms |
| ☒ | ☐ Clinical data |
| ☒ | ☐ Dual use research of concern |

## Methods

| n/a | Involved in the study |
| --- | --- |
| ☒ | ☐ ChIP-seq |
| ☒ | ☐ Flow cytometry |
| ☒ | ☐ MRI-based neuroimaging |
