# Supplementary material for: Reduced dynamic complexity allows structure elucidation of an excited state of KRASG13D
Source: Commun Biol. 2023 Jun 2;6:594. doi: 10.1038/s42003-023-04960-6 (PMC10238545; doi:10.1038/s42003-023-04960-6)
Supplement: Supplementary file 1 — Cornilescu_Peer review file [file 42003_2023_4960_MOESM1_ESM.pdf]

nature portfolio

Peer Review File

Identification and structure elucidation of an excited state in a KRAS G13D oncogenic mutant with reduced dynamic complexity

Open Access This file is licensed under a Creative Commons Attribution 4.0 International License, which permits use, sharing, adaptation, distribution and reproduction in any medium or format, as long as you give appropriate credit to the original author(s) and the source, provide a link to the Creative Commons license, and indicate if changes were made. In the cases where the authors are anonymous, such as is the case for the reports of anonymous peer reviewers, author attribution should be to 'Anonymous Referee' followed by a clear attribution to the source work. The images or other third party material in this file are included in the article's Creative Commons license, unless indicated otherwise in a credit line to the material. If material is not included in the article's Creative Commons license and your intended use is not permitted by statutory regulation or exceeds the permitted use, you will need to obtain permission directly from the copyright holder. To view a copy of this license, visit http://creativecommons.org/licenses/by/4.0/.

Reviewers' comments:

Reviewer #1 (Remarks to the Author):

The paper entitled ”Identification and Structure Elucidation of an Excited State in a KRAS G13D Oncogenic Mutant with Reduced Dynamic Complexity” by Chao et al. aims at characterizing the excited state of oncogenic KRAS-G13D mutant protein bound to GTP analogue GPPNHP by various NMR methods (methyl- and amide-based relaxation dispersion, line shape analysis, 1D 31P-NMR, RDCs) and X-ray crystallography. The authors found that GMPPNP-bound KRAS-G13D exhibits slow timescale (ms-μs) conformational motions as known for GTP-bound forms of other KRAS proteins, however its internal motions in this time scale are more synchronized along the entire protein than that of the wildtype and other mutants. The excited state ensemble is possibly less heterogeneous in the conformational space than the excited states in case of other KRAS proteins. They solved the ground state-structure (state I) of KRAS-G13D bound to GMPPNP by X-ray crystallography with a higher resolution than previously known X-ray structure, and generated the excited state's conformational ensemble using RDC data.

The chosen methods are well suited for the goal of the investigation, the data are nicely presented and analyzed carefully. The conclusions are drawn very logically. The results are novel – as far as I know there has been no suggested structure for the excited state of KRAS-G13D in GTP (or analogue)-bound form, which could be highly interesting as a target for drug design against G13D mutation induced cancers. The thorough characterization of the slow dynamics of KRAS mutants and comparison with the wildtype and other mutants are very important to understand better the RAS-related cancer mechanism. And it is also highly interesting as a good example for general allosteric mechanism for proteins. By Cho et al.'s work one more 'puzzle' in the very complex picture of the conformational landscape of KRAS and its mutants was discovered.

I would have several minor questions and suggestions to the authors, which are not critiques but rather attempts to improve the quality of their nice work:

1. In the Introduction section (page 3) they claim that: ”Although it had been shown that the dynamics of KRAS can be affected by using non-hydrolyzable GTP analogs…”. This was studied carefully in case of HRAS and not KRAS in this paper: Long D, Marshall CB, Bouvignies G, et al.: „A comparative CEST NMR study of slow conformational dynamics of small GTPases complexed with GTP and GTP analogues.” Angew Chem Int Ed Engl. 2013;52(41):10771-10774. doi:10.1002/anie.201305434. To my best knowledge the dynamic properties of the different GTP analogues have not been compared similar way in case of KRAS. And there are some differences in the allosteric lobes (and consequently the dynamics also) between HRAS and KRAS. So it would be probably better to cite this paper and use ”RAS” and not ”KRAS” in the sentence.

2. The continuation of this sentence is: ”…recent dynamic studies using GTP[17], [18] showed that two different minor states of GTP-bound RAS can be produced by using different experimental conditions, highlight the complex dynamics of RAS proteins in solution.” Here [17] reference is also for HRAS, while [18] for KRAS. I think it would be nice to mention the difference in the sentence to avoid confusing HRAS and KRAS.

3. In the Results section in page 5, they state that: ”The conformational exchange rates are reduced relative to GDP-bound KRASG13D, which shows fast and diverse conformational dynamics (Figure S2)”. Interestingly, this finding is in contrast to some recent earlier work which was cited in the Introduction section: in [18] reference (Pálfy G, Menyhárd DK, Ákontz-Kiss H, et al. The Importance of Mg2+ -Free State in Nucleotide Exchange of Oncogenic K-Ras Mutants. Chemistry. 2022;28(59):e202201449. doi:10.1002/chem.202201449) the authors found opposite tendency: GTP-bound (native GTP!) forms exhibit slow motions with larger kex values compared to GDP-bound forms for wt, G12C, G12D and G12V. These experiments were carried out also at 25 °C. What could be the

reason for this difference? Probably the tendency of the backbone (Pálfy et al. results) vs. side-chain dynamics (current study)? It would be useful to have a comparison or discuss this difference too in the paper.

4. In Fig. 1A, Fig. 4A-F and Fig. S4B they should add to the caption which PDB structure they used for visualization: are these from the literature or their own structure?

5. One of the last sentences in page 5 starts like this: ”The overall change in conformational dynamics of KRASG13D in the presence of phosphate is similar to that of KRASWT (Figure S2).” In fact it seems that the authors wanted to refer to the overall change over the temperature change. It would be good to make it clearer what type of overall change they meant.

6. Only a suggestions which the authors could consider: in Fig. 4D and 4E the message would be probably better seen if they show them similarly to Fig. 4F (zoom and closer).

7. In the Discussion section it would be nice to compare their results with the slow dynamics of wildtype and other mutants in a more detailed way to connect their results to the already known data from the literature. For this, the following two reviews could be helpful: Mott HR, Owen D. Allostery and dynamics in small G proteins. Biochem Soc Trans. 2018;46(5):1333-1343. doi:10.1042/BST20170569 and Pálfy G, Menyhárd DK, Perczel A. Dynamically encoded reactivity of Ras enzymes: opening new frontiers for drug discovery. Cancer Metastasis Rev. 2020;39(4):1075-1089. doi:10.1007/s10555-020-09917-3.

Further minor comments related to terminology, typos and figures:

8. In Fig. 1B-E and Fig. S2 in the y axis they used 10^y in brackets which makes no sense. They should give the right value for kex in the y axis.

9. In one of the last sentence in page 11 there is probably a typo: they used RMDS instead of RMSD. The sentence starts like this: ”This contrasts with the recently reported 3.4 Å resolution structure of GMPPNP-bound KRASG13D (PDB: 6E6F; RMDS : 0.612 Å)”.

10. In Fig. 4F there are some symbols in the label of B and C molecules, which should be corrected.

11. In page 14 in the one of the last sentences they refer to Fig. S5, but it should be probably Fig. 5 (main text, not SI): ”This suggested that the crystal structures do not completely represent the solution state of KRASG13D and led us to generate an N=2 ensemble of solution structure models that would fit the RDC restraints within (but not tighter than) the experimental precision, as shown in Figure S5.”

12.In page 16 when describing the RDC vectors, sometimes they use CO for carbonyls but before and after they use C’ for carbonyls. The same terminology throughout the entire paper would be better (in this sentence: ”When selecting only the residues showing the most significant changes in the spatial orientation of the RDC vectors (i.e., N-H, CA-HA, N-COi, N-COi-1 of residues 7,…”).

13. The numbering of Fig. S8 and S9 could be the other way around (Fig. S8 should be S9 and S9 should be S8) considering their first appearance in the text.

14. The numbering of Fig. S10 and S11 could be the other way around (Fig. S10 should be S11 and S11 should be S10) considering their first appearance in the text.

15. In the middle of page 20 NRAS should be written as NRAS and not N-RAS to be consistent in terminology considering that the term of ”KRAS” and ”HRAS” are used (with no hyphen).

16. In page 24 in word D2O number 2 should be written with subscript.

17. In Fig. S4B it is not clear whether the Ile-s shown as spheres are red because their ρRex values are in between 6.5-11.5 s-1 or the color codes are not systematically used in the figure. Other Ile-s are not shown as red spheres.

18. In Fig. S6 and S7 the blue texts in the graphs have poor resolution in the figure which needs to be improved to be similar to Fig. S5.

Overall, the work of Chao et al. presents interesting and novel data acquired by thoroughly carried out NMR and X-ray experiments, which are relevant for the broader audience interested in cancer mechanism as well as protein allostery.

Reviewer #2 (Remarks to the Author):

The manuscript used diverse biophysical experiments to study the localized dynamics of an excited state in a KRAS G13D oncogenic mutant. The study topic is interesting and with biological significance. The results seem to be well supported by the data. Therefore, the reviewer suggests it can be published when the following questions are addressed.
1. On page 5 line 141, the authors claimed that "we found that GMPPNP-bound KRASG13D displays a rare synchronized conformational dynamics behavior connecting the effector and allosteric lobes." Please provide more details about it. What do you mean by synchronized conformational dynamics? Why is it unique for GMPPNP-bound KRASG13D?
2. In Figure S2, the reviewer can not find the γ phosphate, what do you mean "KRASG13D in the presence of γphosphate" on page 5 line145?
3. It is better to draw the structure of PDB:6E6F for comparison. Please give an explanation why 6E6F has a different conformation for Switch I when in the same complexes.
4. Please label the residue Y32 in Figures 4A and 4B for comparison. Please include the PDB numbers in Figure 4 for all deposited structures.
5. In Figure 4F, G13D has a similar conformation with the wt. Then, what is the reason that G13D can affect the function of the RAS protein?
6. Before talking about the result from the RDC experiments, please give some background for the RDC experiment and why it can be used to determine structures. Also, please provide a detailed protocol for RDC calculation, including the restraints and parameters. I suggest including the restraint files and the script in supporting information or depositing them in the public database.
7. Did the authors try to do the MD simulation for the system? Can MD simulation be used to prove the proposed synchronized conformational dynamics?

Reviewer #3 (Remarks to the Author):

The manuscript by Chao et al. utilizes a novel approach to capture and structurally characterize a previously unidentified intermediate state of the oncogenic KRASG13D mutant. Using a novel combination of NMR methods, high-resolution X-Ray crystallographic structures, and isothermal titration calorimetry, this study provided convincing evidence that GMPPNP-bound KRASG13D exists in an intermediate state different from that of GMPPNP-bound KRASWT, GDP-KRASG13D or RAF-RBD-bound KRASG12R.

Methyl relaxation dispersion experiments revealed GMPPNP-bound KRASG13D exhibits unique and highly synchronized conformational dynamics connecting both the effector and allosteric lobes compared to GMPPNP-bound KRASWT and two oncogenic KRAS mutants, KRASG12D and KRASQ61H.

Methyl and 31P NMR spectra of KRASWT, KRASG13D, and the “State 1” stabilizing mutation T35S revealed a two-state population ensemble, which appears to be unique to the KRASG13D mutation. G12D and Q61H were also screened in this study but failed to exhibit behavior similar to G13D.

X-Ray crystallography structures of KRASG13D (both GMPPNP and GDP-bound) have been previously published (Johnson et al, 2019). However, this manuscript picks up nicely where those studies left off by providing higher resolution detail about key residues in the active GMPPNP-bound form (the previous G13D structure was 3.4 angstroms, while the new structure is reported at 1.2 angstroms). Furthermore, a novel complex structure of a GMPPNP-bound KRASG13D and the Raf-RBD was solved (2.0 angstroms). These structures were analyzed alongside a previous KRASG13D structure (Johnson et al., 2019) and an intermediate KRASG13D structure derived using residual dipolar couplings. The analysis provided great insight into the two-state ensemble of KRASG13D and the dynamics associated with the KRASG13D mutation. The intermediate structure also revealed a small hydrophobic pocket near A130, which could be exploited for drug discovery efforts looking to target KRASG13D-driven cancers.

Overall, I think this manuscript does an excellent job of building on previous work related to characterizing the dynamics and structural uniqueness of the oncogenic KRASG13D mutant. Furthermore, it provides valuable information that can be useful in helping target this oncogenic KRAS mutant and possibly others. I recommend this manuscript for publication.

Minor Points

1. It would have been nice to have more information or discussion related to this pocket, including the size of the pocket or a more detailed image of the pocket.

2. The X-Ray crystallography statistics look good, and the structural analysis was laid out logically (loved Figure 4). I thought all the experiments were designed well. I was hoping for more insight from the ITC results. I would have liked more discussion about the differences in binding affinities, given the KRASG12R,A130I mutant had a similar population distribution compared to the KRASG12R,A130V and KRASG12R,A130L mutants.

3. Fig 4: A PDB accession code is missing for the GDP-bound KRASG13D structure (I assume you are referencing 6E6G?). I would also suggest that you reference PDB accession codes in the figure legend of Fig. 4.

4. Line 54-56: I suggest you also reference the nucleotide-exchange data from (Johnson et al (2019). Isoform-Specific Destabilization of the Active Site Reveals a Molecular Mechanism of Intrinsic Activation of KRas G13D)) for KRASG13D and HRASG13D, in addition to the references you already listed (Reference #4 and #5).

5. Line 420: KRASG13D is listed as “KRASG13D,” rather than listing G13D in superscript

6. Line 516: When you state that the excited (intermediate) state is distinct from the known dominant state 1 and 2 in KRASWT and “most of its mutants”, are you only referring to G12D and Q61H, or are there other mutants that have been analyzed?

ITC

1. Missing: number of biological replicates per sample (N) and no statistical analysis is reported
- No error is given for the ITC/thermo data in Supplementary Fig. 11
- There is error reported with the binding affinities in Supp. Table 3, but how was it calculated and what about error for your other thermodynamic parameters?

2. The “Methods” section only lists the ITC buffer with 150 mM NaCl and doesn’t mention the 300 mM NaCl ITC buffer used in the supplementary section.

3. N-value is missing for all ITC experiments

4. What temperature did you collect your ITC experiments at?

5. Your KRASG13D ITC data shows a larger enthalpic term compared to entropy, while previous ITC data (Johnson et al, 2019) shows a more even split between these two terms

Point-by-point response:

We appreciate the extensive and thoughtful insights from the reviewers that have led to a significantly improved manuscript. We thank the reviewers for their time and efforts. In the sections that follow, the reviewer's points and concerns are in black, our response is in *italic blue*, and the text incorporated in the revised manuscript is shown in *italic bright blue*.

Reviewer #1 (Remarks to the Author):

The paper entitled "Identification and Structure Elucidation of an Excited State in a KRAS G13D Oncogenic Mutant with Reduced Dynamic Complexity" by Chao et al. aims at characterizing the excited state of oncogenic KRAS-G13D mutant protein bound to GTP analogue GPPNHP by various NMR methods (methyl- and amide-based relaxation dispersion, line shape analysis, 1D 31P-NMR, RDCs) and X-ray crystallography. The authors found that GMPPNP-bound KRAS-G13D exhibits slow timescale (ms-µs) conformational motions as known for GTP-bound forms of other KRAS proteins, however its internal motions in this time scale are more synchronized along the entire protein than that of the wildtype and other mutants. The excited state ensemble is possibly less heterogeneous in the conformational space than the excited states in case of other KRAS proteins. They solved the ground state-structure (state I) of KRAS-G13D bound to GMPPNP by X-ray crystallography with a higher resolution than previously known X-ray structure, and generated the excited state's conformational ensemble using RDC data.

The chosen methods are well suited for the goal of the investigation, the data are nicely presented and analyzed carefully. The conclusions are drawn very logically. The results are novel – as far as I know there has been no suggested structure for the excited state of KRAS-G13D in GTP (or analogue)-bound form, which could be highly interesting as a target for drug design against G13D mutation induced cancers. The thorough characterization of the slow dynamics of KRAS mutants and comparison with the wildtype and other mutants are very important to understand better the RAS-related cancer mechanism. And it is also highly interesting as a good example for general allosteric mechanism for proteins. By Cho et al.'s work one more 'puzzle' in the very complex picture of the conformational landscape of KRAS and its mutants was discovered.

We appreciate that the reviewer recognizes our work's significance and are grateful for their insightful suggestions, comments, and questions.

I would have several minor questions and suggestions to the authors, which are not critiques but rather attempts to improve the quality of their nice work:

1. In the Introduction section (page 3) they claim that: "Although it had been shown that the dynamics of KRAS can be affected by using non-hydrolyzable GTP analogs…". This was studied carefully in case of HRAS and not KRAS in this paper: Long D, Marshall CB, Bouvignies G, et al.: „A comparative CEST NMR study of slow conformational dynamics of small GTPases complexed with GTP and GTP analogues." Angew Chem Int Ed Engl. 2013;52(41):10771-10774.

doi:10.1002/anie.201305434. To my best knowledge the dynamic properties of the different GTP analogues have not been compared similar way in case of KRAS. And there are some differences in the allosteric lobes (and consequently the dynamics also) between HRAS and KRAS. So it would be probably better to cite this paper and use ”RAS” and not ”KRAS” in the sentence.

The reviewer is right, and we have now corrected the inaccuracy. We strongly agree that the sequence differences in the allosteric lobe of HRAS would likely translate into distinct dynamic behaviors. We added the reference to the HRAS/KRAS study of non-hydrolyzable GTP analogs and used "the HRAS isoform" instead of "KRAS" in the Introduction section (page 3, line 83).

2. The continuation of this sentence is: ”…recent dynamic studies using GTP[17], [18] showed that two different minor states of GTP-bound RAS can be produced by using different experimental conditions, highlight the complex dynamics of RAS proteins in solution.” Here [17] reference is also for HRAS, while [18] for KRAS. I think it would be nice to mention the difference in the sentence to avoid confusing HRAS and KRAS.

Indeed, the distinction is needed, and we modified the text on page 3 (lines 82-88) to specify the isoforms used in those studies:

"It had been shown that the dynamics of the HRAS isoform can be affected by using non-hydrolyzable GTP analogs,[18] and recent dynamic studies using GTP with HRAS[17] and with KRAS[19] showed that two different minor states of GTP-bound RAS can be produced under different experimental conditions. Interestingly, although HRAS and KRAS proteins have an identical primary sequence in the effector lobe, they display different structural features in the reported minor states, highlighting the complex dynamics of RAS proteins in solution."

3. In the Results section in page 5, they state that: ”The conformational exchange rates are reduced relative to GDP-bound KRASG13D, which shows fast and diverse conformational dynamics (Figure S2)”. Interestingly, this finding is in contrast to some recent earlier work which was cited in the Introduction section: in [18] reference (Pálfy G, Menyhárd DK, Ákontz-Kiss H, et al. The Importance of Mg2+ -Free State in Nucleotide Exchange of Oncogenic K-Ras Mutants. Chemistry. 2022;28(59):e202201449. doi:10.1002/chem.202201449) the authors found opposite tendency: GTP-bound (native GTP!) forms exhibit slow motions with larger kex values compared to GDP-bound forms for wt, G12C, G12D and G12V. These experiments were carried out also at 25 °C. What could be the reason for this difference? Probably the tendency of the backbone (Pálfy et al. results) vs. side-chain dynamics (current study)? It would be useful to have a comparison or discuss this difference too in the paper.

The reviewer has a great point. The differences stem from the different experimental conditions, and the different results reflect distinct biological functions of KRAS proteins. In the Palfy et al. study, to stabilize the highly unstable Mg^{2+} free state, excessive

amounts of nucleotides were added to the buffer in the Palfy et al. study. Consequently, the dynamic equilibria (or free energy landscape) were altered, making the Mg^{2+} free state the second dominant population. Under these conditions, relaxation dispersion experiments primarily detect the conformational exchange between the Mg2+ bound state (the dominant state) and the Mg^{2+} free state (the second dominant state). The Mg^{2+} free state detected by relaxation dispersion experiments was further confirmed by comparing chemical shift differences with those observed after adding 10 mM EDTA in the buffer without Mg^{2+}. In the Palfy et al. study, the exchange rates between these two conformations were reported as the rate-limiting step of the intrinsic nucleotide exchange. In contrast, in all our experiments, including those published previously (doi.org/10.1021/jacs.2c00007), the buffer does not contain additional nucleotides (nor EDTA).

To understand the conformational dynamics relevant to the interaction between KRAS and its effectors, and to identify conformations potentially relevant to drug discovery, it is important to minimize the dynamic complications induced by the intrinsic nucleotide exchange process of RAS proteins. Therefore, under our experimental conditions, the relaxation dispersion techniques report on the dynamics relevant to the interaction between KRAS and its effectors. In this context, our dynamic data here and in our previous report (doi.org/10.1021/jacs.2c00007) demonstrate the plasticity of KRAS proteins when interacting with the effectors. To emphasize these points, we added the following to the discussion section (lines 480-483):
"Although two minor states and the conformational exchanges involved in intrinsic nucleotide exchange have been revealed by GTP-bound RAS proteins,[17, 19] the results of this and our previous study[16] using the non-hydrolyzable GTP analog GMPPNP, demonstrate the plasticity of KRAS proteins when interacting with their effectors instead."

4. In Fig. 1A, Fig. 4A-F and Fig. S4B they should add to the caption which PDB structure they used for visualization: are these from the literature or their own structure?

We used our newly determined crystal structures and have now specified their PDB IDs in all figure captions.

5. One of the last sentences in page 5 starts like this: "The overall change in conformational dynamics of KRASG13D in the presence of phosphate is similar to that of KRASWT (Figure S2)." In fact it seems that the authors wanted to refer to the overall change over the temperature change. It would be good to make it clearer what type of overall change they meant.

We are referring to the contrast between the slower exchange rates in the GMPPNP-bound state relative to the GDP-bound state. To clarify this, we modified the text (lines 145-147) to read:
*"This overall trend of reduced conformational exchange rates of $KRAS^{G13D}$ in the presence of the γphosphate is similar to that of $KRAS^{WT}$ (**Supplementary Fig. 2**)."*

6. Only a suggestions which the authors could consider: in Fig. 4D and 4E the message would be probably better seen if they show them similarly to Fig. 4F (zoom and closer).

We prefer to maintain the current "zoomed-out" version of Fig. 4D and 4E to show that the overlaid KRAS structures have similar conformation outside the switch regions. Panel 4F already contains the zoomed-in views of both Fig. 4D and 4E.

7. In the Discussion section it would be nice to compare their results with the slow dynamics of wildtype and other mutants in a more detailed way to connect their results to the already known data from the literature. For this, the following two reviews could be helpful: Mott HR, Owen D. Allostery and dynamics in small G proteins. Biochem Soc Trans. 2018;46(5):1333-1343. doi:10.1042/BST20170569 and Pálfy G, Menyhárd DK, Perczel A. Dynamically encoded reactivity of Ras enzymes: opening new frontiers for drug discovery. Cancer Metastasis Rev. 2020;39(4):1075-1089. doi:10.1007/s10555-020-09917-3.

The focus of this manuscript is to experimentally determine the structure of a minor state of KRAS proteins in solution while simply using the dynamic data from methyl relaxation dispersion experiments as a way to identify such a state. It will be the subject of a separate paper to extensively review and compare all our published and unpublished dynamics data with that in other published studies. Thus, it is beyond the scope of the current manuscript to include an in-depth analysis of $KRAS^{WT}$ and mutant dynamics.

Further minor comments related to terminology, typos and figures:

8. In Fig. 1B-E and Fig. S2 in the y axis they used 10^y in brackets which makes no sense. They should give the right value for kex in the y axis.

Corrected; thank you for the suggestion.

9. In one of the last sentence in page 11 there is probably a typo: they used RMDS instead of RMSD. The sentence starts like this: "This contrasts with the recently reported 3.4 Å resolution structure of GMPPNP-bound KRASG13D (PDB: 6E6F; RMDS : 0.612 Å)".

Corrected.

10. In Fig. 4F there are some symbols in the label of B and C molecules, which should be corrected.

It is unclear to us what symbols need correction: the arrows in the legend underneath the Fig. 4F panel? Or the B and C letters themselves, inside the panel? Or something else? The A, B, and C letters are visual guides illustrating differences in side-chain positions between the molecules overlaid; they do not designate molecules.

11. In page 14 in the one of the last sentences they refer to Fig. S5, but it should be probably Fig. 5 (main text, not SI): ”This suggested that the crystal structures do not completely represent the solution state of KRASG13D and led us to generate an N=2 ensemble of solution structure models that would fit the RDC restraints within (but not tighter than) the experimental precision, as shown in Figure S5.”

On page 14, we refer to the fact that we adjusted the Xplor-NIH force factors of each set of experimental RDC restraints to avoid overfitting. This is illustrated in Supplementary Fig. 5, in which the RMSD between the observed and back-calculated RDCs using the RDC refined structures matches approximately the precision of the measured RDCs. This is also described in more detail on pg. 15.

12.In page 16 when describing the RDC vectors, sometimes they use CO for carbonyls but before and after they use C’ for carbonyls. The same terminology throughout the entire paper would be better (in this sentence: ”When selecting only the residues showing the most significant changes in the spatial orientation of the RDC vectors (i.e., N-H, CA-HA, N-COi, N-COi-1 of residues 7,…”).

We replaced CO with C’ (lines 379-380); thank you for the suggestion.

13. The numbering of Fig. S8 and S9 could be the other way around (Fig. S8 should be S9 and S9 should be S8) considering their first appearance in the text.

We swapped Supplementary Fig. 8 and Supplementary Fig. 9, thank you for noticing.

14. The numbering of Fig. S10 and S11 could be the other way around (Fig. S10 should be S11 and S11 should be S10) considering their first appearance in the text.

We also swapped Supplementary Fig. 10 and Supplementary Fig. 11.

15. In the middle of page 20 NRAS should be written as NRAS and not N-RAS to be consistent in terminology considering that the term of ”KRAS” and ”HRAS” are used (with no hyphen).

We eliminated the cited monobody and DARPins studies from the discussion on pp. 20-21 owing to recent critiques of their conclusions: *https://www.sciencedirect.com/science/article/pii/S109727652300165X*

16. In page 24 in word D2O number 2 should be written with subscript.

Corrected (line 625).

17. In Fig. S4B it is not clear whether the Ile-s shown as spheres are red because their ρRex values are in between 6.5-11.5 s-1 or the color codes are not systematically used in the figure.

Other Ile-s are not shown as red spheres.

We thank the reviewer for noticing this. In Supplementary Fig. 4B, we wanted to present a complete picture of the conformational dynamics of KRAS^{G13D} distributed throughout the whole protein. We accidentally colored the methyl groups with a color existent in the color scale for amide groups. Still, the relaxation dispersion data from different chemical groups are not comparable due to the different sizes of their chemical shift differences. We have now corrected Supplementary Fig. 4B using blue (line 981) for all the methyl groups with a synchronized motion.

18. In Fig. S6 and S7 the blue texts in the graphs have poor resolution in the figure which needs to be improved to be similar to Fig. S5.

The reviewer may have received a low-resolution converted file. The manuscript contains postscript (vector graphics) for Supplementary Fig. 5, 6, and 7 (infinite resolution); the blue text is identical in all of them.

Overall, the work of Chao et al. presents interesting and novel data acquired by thoroughly carried out NMR and X-ray experiments, which are relevant for the broader audience interested in cancer mechanism as well as protein allostery.

We again thank the reviewer for the thorough analysis and suggestions, which have improved our manuscript.

Reviewer #2 (Remarks to the Author):

The manuscript used diverse biophysical experiments to study the localized dynamics of an excited state in a KRAS G13D oncogenic mutant. The study topic is interesting and with biological significance. The results seem to be well supported by the data. Therefore, the reviewer suggests it can be published when the following questions are addressed.

We thank the reviewer for appreciating the significance of our study, and we are grateful for their insightful suggestions, comments, and questions.

1. On page 5 line 141, the authors claimed that “we found that GMPPNP-bound KRASG13D displays a rare synchronized conformational dynamics behavior connecting the effector and allosteric lobes.” Please provide more details about it. What do you mean by synchronized conformational dynamics? Why is it unique for GMPPNP-bound KRASG13D?

By synchronized conformational dynamics, we mean that the conformational exchange rates for all methyl reporters (distributed in both effector and allosteric lobes of the GMPPNP-bound KRAS^{G13D}) are very similar and that they change in sync with the temperature, as shown in Fig. 1D. The slopes of their linearized Arrhenius dependencies

are very similar and virtually overlap, i.e., they have the same conformational exchange rates at all the temperatures measured. This contrasts with the experimental results obtained for $KRAS^{WT}$ and other KRAS mutants (Fig. 1B, 1C, 1E).

2. In Figure S2, the reviewer can not find the γ phosphate, what do you mean "KRASG13D in the presence of γphosphate" on page 5 line145?

The presence or absence of the γ phosphate is just another way of specifying the nucleotide bound to KRAS, with GDP having only α and β phosphate atoms, while only GMPPNP (or GTP) has the 3rd γ phosphate atom. In other words, the presence of the γ phosphate is a synonym for the GMPPNP-bound state of KRAS.

3. It is better to draw the structure of PDB:6E6F for comparison. Please give an explanation why 6E6F has a different conformation for Switch I when in the same complexes.

6E6F and the structure we solved (8EBZ) were crystallized in different conditions, with different crystal forms showing different crystal contacts. Since Switch I is flexible in state 1, it adopted different conformations in the two crystal forms. We have now inserted this explanation in the text by replacing (on lines 277-278) "two snapshots" with "the dynamic Switch I is captured in two different snapshots as observed in two different crystal forms."

4. Please label the residue Y32 in Figures 4A and 4B for comparison. Please include the PDB numbers in Figure 4 for all deposited structures.

In Figures 4A and 4B, we first represented the States 1 and 2 of GMPPNP-bound $KRAS^{G13D}$ to highlight the Switch regions relative to the nucleotide position. The same structures are then overlaid in Fig. 4D, with residue Y32 labeled, allowing the comparison of the Y32 position requested by the reviewer. We have now included in all figure captions the PDB IDs of all the crystal structures shown.

5. In Figure 4F, G13D has a similar conformation with the wt. Then, what is the reason that G13D can affect the function of the RAS protein?

While $KRAS^{G13D}$ and $KRAS^{WT}$ have similar conformation in their crystal structures, GDP-bound $KRAS^{G13D}$ has higher SOS-dependent and SOS-independent GDP/GTP exchange rates, respectively, as mentioned in the introduction. Moreover, like other oncogenic KRAS mutants, $KRAS^{G13D}$ is insensitive to GTP hydrolysis by GAPs (except for NF1), due to the steric clash between the mutated residue and the arginine finger in GAP. The higher GDP to GTP exchange rate and the lower GTP hydrolysis rate result in more $KRAS^{G13D}$ in the GTP-bound (active) state compared to $KRAS^{WT}$, which makes this mutation oncogenic.

Strikingly, KRAS^{G13D} and KRAS^{WT} have different compositions of substates (as shown in the ^{31}P spectra in Fig. 3) and different dynamics (as shown in Fig. 1), which single crystal structure snapshots cannot capture. These subtle differences, corresponding to functional differences, motivated us to examine their dynamics and obtain the structure of the observed intermediate state of G13D.

6. Before talking about the result from the RDC experiments, please give some background for the RDC experiment and why it can be used to determine structures. Also, please provide a detailed protocol for RDC calculation, including the restraints and parameters. I suggest including the restraint files and the script in supporting information or depositing them in the public database.

We did introduce the RDC method on pg. 4, but we agree with the reviewer that more info on the RDC experiments, restraints, and parameters may be beneficial. To that extent, we added references to two relevant NMR papers about conformational ensemble refinement using RDCs in the Introduction on pg. 4 (line 114). Furthermore, we added a reference to a good review of the precise structural and dynamics information provided by experimental RDCs on pg. 14 (line 332) of the Results section. Describing RDC experiments in even more detail in the current manuscript would push it beyond the recommended word limit for Communications Biology articles, so we chose to refer to the vast literature sources instead (many of them included in the review article above).

7. Did the authors try to do the MD simulation for the system? Can MD simulation be used to prove the proposed synchronized conformational dynamics?

We did not try to do the MD simulation because, while there are several studies of KRAS dynamics using MD simulations and we cited (lines 69-70) a good review that discusses them (Pantsar T. The current understanding of KRAS protein structure and dynamics. Comput. Struct. Biotechnol. J. 18, 189-198, 2020). We feel that using experimental restraints (RDCs) in an Xplor-NIH simulated annealing protocol (which includes MD stages restrained by experimental data) provides superior results that can be cross-validated against the restraints used.

We believe that the synchronized conformational dynamics is proved by the relaxation dispersion data, which shows a specific and striking synchronized behavior of the reporting methyl groups (Fig. 1) and by the presence of a corresponding single distinct intermediate state visible in methyl, amide, and ^{31}P NMR spectra.

Reviewer #3 (Remarks to the Author):

The manuscript by Chao et al. utilizes a novel approach to capture and structurally characterize a previously unidentified intermediate state of the oncogenic KRASG13D mutant. Using a novel combination of NMR methods, high-resolution X-Ray crystallographic structures, and

isothermal titration calorimetry, this study provided convincing evidence that GMPPNP-bound KRASG13D exists in an intermediate state different from that of GMPPNP-bound KRASWT, GDP-KRASG13D or RAF-RBD-bound KRASG12R.

Methyl relaxation dispersion experiments revealed GMPPNP-bound KRASG13D exhibits unique and highly synchronized conformational dynamics connecting both the effector and allosteric lobes compared to GMPPNP-bound KRASWT and two oncogenic KRAS mutants, KRASG12D and KRASQ61H. Methyl and 31P NMR spectra of KRASWT, KRASG13D, and the "State 1" stabilizing mutation T35S revealed a two-state population ensemble, which appears to be unique to the KRASG13D mutation. G12D and Q61H were also screened in this study but failed to exhibit behavior similar to G13D.

X-Ray crystallography structures of KRASG13D (both GMPPNP and GDP-bound) have been previously published (Johnson et al, 2019). However, this manuscript picks up nicely where those studies left off by providing higher resolution detail about key residues in the active GMPPNP-bound form (the previous G13D structure was 3.4 angstroms, while the new structure is reported at 1.2 angstroms). Furthermore, a novel complex structure of a GMPPNP-bound KRASG13D and the Raf-RBD was solved (2.0 angstroms). These structures were analyzed alongside a previous KRASG13D structure (Johnson et al., 2019) and an intermediate KRASG13D structure derived using residual dipolar couplings. The analysis provided great insight into the two-state ensemble of KRASG13D and the dynamics associated with the KRASG13D mutation. The intermediate structure also revealed a small hydrophobic pocket near A130, which could be exploited for drug discovery efforts looking to target KRASG13D-driven cancers.

Overall, I think this manuscript does an excellent job of building on previous work related to characterizing the dynamics and structural uniqueness of the oncogenic KRASG13D mutant. Furthermore, it provides valuable information that can be useful in helping target this oncogenic KRAS mutant and possibly others. I recommend this manuscript for publication.

We are grateful for the reviewer's appreciation of our study and their insightful suggestions, comments, and questions.

Minor Points

1. It would have been nice to have more information or discussion related to this pocket, including the size of the pocket or a more detailed image of the pocket.

Since our structural restraints are limited to backbone RDCs, the structural ensemble of the intermediate state has a lower side chain resolution and therefore cannot provide more precise structural details of that pocket. Nonetheless, the presence of the pocket is confirmed by the systematically introduced mutations to A130 and by the ^{31}P NMR spectra showing altered population distributions induced by the mutations, as predicted based on our RDC-refined structural ensemble.

In addition, we replaced the following sentence in the Discussion section (lines 487-489):

"Finally, we introduced secondary mutations to A130 to further validate the structure of the intermediate state and the dynamic coupling between the effector and allosteric lobes."

with:
*"Finally, we introduced a series of additional mutations at A130 to validate the presence of a small hydrophobic pocket in the allosteric lobe by monitoring the population shifts in the effector lobe using ^{31}P NMR spectra (**Fig. 6C** and **Supplementary Fig. 11**)."*

2. The X-Ray crystallography statistics look good, and the structural analysis was laid out logically (loved Figure 4). I thought all the experiments were designed well. I was hoping for more insight from the ITC results. I would have liked more discussion about the differences in binding affinities, given the KRASG12R,A130I mutant had a similar population distribution compared to the KRASG12R,A130V and KRASG12R,A130L mutants.

We are glad to know that the reviewer liked our structural analysis in Figure 4. As suggested, we have now added more details and insights from the ITC results. We supplemented the Discussion section (lines 489-501) with:
*"Even though all these double mutants of KRAS display similar conformational population distribution, they have different binding affinities to the RAF1-RBD effector, as shown by ITC data (**Supplementary Fig. 10**). We observed a similar situation in our previous study,[16] where three different mutations stabilizing the State I conformation in KRAS displayed different dynamics coupled with different binding affinities to RAF1-RBD. The result agrees with an earlier study of the same mutations in HRAS.[32] Our ITC data and previous results[16, 32] suggest that the population distribution of RAS conformations in solution is not the sole factor affecting the binding of RAS proteins to the effectors. On the other hand, our ^{31}P spectra on $KRAS^{G13D}$ (**Fig. 3**) and a previous transient kinetic study[25] support that binding RAS proteins to their effectors involves both conformational selection and induced fit. The induced fit from the intermediate state to the bound state and additional unpredicted entropic contributions could explain the variation in the binding affinities of different KRAS double mutants to RAF1-RBD."*

3. Fig 4: A PDB accession code is missing for the GDP-bound KRASG13D structure (I assume you are referencing 6E6G?). I would also suggest that you reference PDB accession codes in the figure legend of Fig. 4.

The GDP-bound $KRAS^{G13D}$ structure came from the higher resolution PDB 4TQA. All the PDB codes are now inserted in the figure legends.

4. Line 54-56: I suggest you also reference the nucleotide-exchange data from (Johnson et al (2019). Isoform-Specific Destabilization of the Active Site Reveals a Molecular Mechanism of

Intrinsic Activation of KRas G13D)) for KRASG13D and HRASG13D, in addition to the references you already listed (Reference #4 and #5).

Reference added in that context as well (on lines 51 and 53); thank you for the suggestion.

5. Line 420: KRASG13D is listed as "KRASG13D," rather than listing G13D in superscript

Corrected (now on line 426).

6. Line 516: When you state that the excited (intermediate) state is distinct from the known dominant state 1 and 2 in KRASWT and "most of its mutants", are you only referring to G12D and Q61H, or are there other mutants that have been analyzed?

We are indeed only referring to G12D and Q61H and have replaced "in $KRAS^{WT}$ and most of its mutants" with "in $KRAS^{WT}$ and its G12D and Q61H mutants" (now on line 539).

ITC

1. Missing: number of biological replicates per sample (N) and no statistical analysis is reported
- No error is given for the ITC/thermo data in Supplementary Fig. 11

Thank you for pointing this out. We have now added the errors in Supplementary Fig. 10 (renumbered from Supplementary Fig. 11) and more details in the legend of Supplementary Table 3, which summarizes all the ITC measurements to read:

"Thermodynamics parameters were calculated from a single ITC experiment (n=1) with 19-data points. Errors in the K_D and ΔH values correspond to the standard error of fitting."

- There is error reported with the binding affinities in Supp. Table 3, but how was it calculated and what about error for your other thermodynamic parameters?

We have now added the error in ΔH values in Supplementary Table 3. We have also included the details about how they were calculated in the legend of Supplementary Table 3.

2. The "Methods" section only lists the ITC buffer with 150 mM NaCl and doesn't mention the 300 mM NaCl ITC buffer used in the supplementary section.

Thank you for spotting that; we have now also mentioned the 300 mM NaCl ITC buffer in the Methods section (line 694).

3. N-value is missing for all ITC experiments

We have now added this detail in Supplementary Table 3.

4. What temperature did you collect your ITC experiments at?

The ITC measurements were performed at 25 °C, and we have now specified that in the ITC Measurements section of the Methods (line 698).

5. Your KRASG13D ITC data shows a larger enthalpic term compared to entropy, while previous ITC data (Johnson et al, 2019) shows a more even split between these two terms

The difference in the split between the enthalpic and entropic terms likely reflects the differences in the ITC buffers used, especially the presence of a detergent in the buffer used by Johnson et al. The buffer used in this study is 20 mM HEPES (pH 7.4), 300 mM NaCl, 5 mM $MgCl_2$, 1mM TCEP, while Johnson et al., 2019 used 20 mM HEPES (pH 7.5), 150 mM NaCl, 10 mM $MgCl_2$, 0.5% (w/v) n-octyl-β-D-glucopyranoside, and 0.5 mM Tris-(2-carboxyethyl)phosphine. Additionally, we used the RAF1-RBD protein that spans from 52 – 131 amino acids and KRAS4b from 1 - 169 amino acids, but Johnson et al., 2019 have used RAF1-RBD protein that spans from 51 – 131 amino acids and KRAS4b protein from 1- 166 amino acids.

REVIEWERS' COMMENTS:

Reviewer #1 (Remarks to the Author):

I accept all of the answers of the authors.

I have only a minor comment on their answer for the 3rd point, which I generally accept as well but I do not agree with them completely for the following reasons.
In Palfy et al.'s work it is clear that they used no high nucleotide access for studying GDP-bound KRAS, while did use a large GTP-access only for GTP-bound forms. Furthermore, the excited state of the slow exchange of GDP-bound form is the Mg2+-free GDP-bound "transition state" between GDP- and GTP-bound states during nucleotide exchange, while the slow exchange of GTP-bound state is more related to the well-known "state 1" and "state 2" (both are GTP- and Mg2+-bound, but active or inactive conformation according to their ability to interact with effector proteins). We can assume that in the authors work (current and previous ones they cited) they have also two different excited states for the GDP- and GMPPNP-bound forms. The result of the kex of the two studies' CPMG measurements are comparable because the kex value is a rate constant (kex = k1 + k-1) which is independent of the concentration of the reactants. The reaction rate equals to the product of the rate constant and the reactants' concentrations, therefore the reaction rate is indeed not comparable, but this is not true for the rate constants if only the concentrations are different. In other words, Palfy et al. slowed down the entire process of GTP-hydrolysis by accelerating the nucleotide exchange rate to increase the population of GTP-bound state in the system for a longer time. Doing this does not affect the rate constant of the reaction, only the final reaction rate.
I think that the difference between the current and Palfy et al.'s studies is more likely originated from other reasons. One of the could be the different method: the authors studied the side chain dynamics through methyl-CPMG, while Palfy et al. studied the backbone dynamics. Since the system is more complex than a simple two-state system, the fitting using the two-state model will not result in an accurate kex value for either approach. Another possible reason could be that the authors used GMPPNP, while Palfy et al. used native GTP, and the nucleotide analogue has indeed influence on the dynamic properties on RAS proteins (see ref 18), which reflected in the kex values too.
Although I do not fully agree with the authors in this point, this question is not very significant in terms of the message of their manuscript. In my point of view, they can decide whether they would like to address any comments on the above-mentioned question in the final version of their manuscript.

Reviewer #2 (Remarks to the Author):

Remarks: The authors responded to the reviewer's comments in a satisfactory manner.
Correction to be addressed:
Although the study focuses on the RAS protein, a number of studies work on the RAS gene regulation, especially for KRAS G-quadruplex. It is better to briefly introduce the KRAS G-quadruplex targeting drug discovery in the introduction or discussion part with some newly published papers to broaden the general interest.

Reviewer #3 (Remarks to the Author):

I appreciated the author's responses to the initial review. All of my questions and concerns were addressed. I approve of all the changes that were made to the manuscript and recommend it for publication in Communications Biology.
